# Bisphenols as Environmental Triggers of Thyroid Dysfunction: Clues and Evidence

**DOI:** 10.3390/ijerph17082654

**Published:** 2020-04-13

**Authors:** Francesca Gorini, Elisa Bustaffa, Alessio Coi, Giorgio Iervasi, Fabrizio Bianchi

**Affiliations:** Institute of Clinical Physiology, National Research Council, 56124 Pisa, Italy; elisa.bustaffa@ifc.cnr.it (E.B.); alessio.coi@ifc.cnr.it (A.C.); iervasi@ifc.cnr.it (G.I.); fabrizio.bianchi@ifc.cnr.it (F.B.)

**Keywords:** Bisphenol A, bisphenols, endocrine disruptors, thyroid hormones, thyroid cancer

## Abstract

Bisphenols (BPs), and especially bisphenol A (BPA), are known endocrine disruptors (EDCs), capable of interfering with estrogen and androgen activities, as well as being suspected of other health outcomes. Given the crucial role of thyroid hormones and the increasing incidence of thyroid carcinoma in the last few decades, this review analyzes the effects of BPS on the thyroid, considering original research in vitro, in vivo, and in humans published from January 2000 to October 2019. Both in vitro and in vivo studies reported the ability of BPs to disrupt thyroid function through multiple mechanisms. The antagonism with thyroid receptors (TRs), which affects TR-mediated transcriptional activity, the direct action of BPs on gene expression at the thyroid and the pituitary level, the competitive binding with thyroid transport proteins, and the induction of toxicity in several cell lines are likely the main mechanisms leading to thyroid dysfunction. In humans, results are more contradictory, though some evidence suggests the potential of BPs in increasing the risk of thyroid nodules. A standardized methodology in toxicological studies and prospective epidemiological studies with individual exposure assessments are warranted to evaluate the pathophysiology resulting in the damage and to establish the temporal relationship between markers of exposure and long-term effects.

## 1. Introduction

Thyroid hormones (THs) play a critical role in the regulation of physical development, somatic growth, metabolism, and energy provision and are essential for normal brain development in humans [1]. Thus, any interference with THs status and signaling during development may have an impact on physical health, and can be associated with neurological deficits and even irreversible mental retardation in the case of severe maternal TH deficiency [2]. Meanwhile, thyroid cancer (TC) incidence rates have been rising in many western countries, including the United States where the incidence increased 3.6% per year during 1974 to 2013 [3]. TC is the most common endocrine malignancy, and by 2030, it is estimated to become the fourth leading cancer diagnosis in the United States [4]. Papillary thyroid cancer (PTC), in particular, is the most frequent histotype with a typically excellent prognosis, accounting for 70% to 90% of well-differentiated thyroid malignancies, and though over diagnosis of small tumors is thought to contribute significantly to the increase in incidence, PTC incidence has significantly increased for every stage and tumor size category [3]. The etiology of TC is multifactorial and the proposed risk factors in the literature include sex, family history of TC, radiation exposure, excess weight, iodine intake, and dietary habits [5]. Although the thyroid is characterized by a low proliferation index, it is particularly susceptible to environmental chemicals that may contribute to the increasing incidence of TC [6].

According to a recent statement by the Endocrine Society, endocrine disrupting chemicals (EDCs) are defined as single exogenous agents or mixture of compounds capable of interfering with any aspect of hormone action, from the synthesis to the transport, catabolism, and elimination of the hormones produced [1,7]. One of the characteristics of many EDCs is a nonmonotonic dose response that replicates hormone characteristics; thus, a proportionally greater effect is observed at low doses than at high doses because of hormone receptor saturation and overstimulation that decrease the response [7,8]. Increasing evidence has shown the ability of several EDCs (e.g., polybrominated diphenyl ethers, polychlorinated biphenyls, pesticides, and phthalates) to exhibit thyroid disrupting activities in animals and, with some inconsistencies, in humans [1,9]. Interference of environmental chemicals with thyroid function can occur at multiple levels including, among others, toxicity at the thyroid gland, disturbance of THs synthesis, secretion and metabolism, competitive binding with the TH binding proteins, and interaction with thyroid hormone receptors (TRs) [1,10].

Bisphenol A (BPA; 2,2-bis(4-hydroxyphenyl)propane) is described as an EDC able to interact with human estrogen receptors (ERs) [11]. In rodent models, a variety of effects was observed in estrogen-target organs (e.g., brain, mammary gland, ovary, and uterus) following exposure to BPA at or below the lowest observed adverse effect level (LOAEL) during prenatal and neonatal periods [12]. Changes in one of the target organs can lead to secondary alterations in bone, adipose tissue, cardiovascular tissue, and the immune system [13]. In humans, increased levels of BPA were associated with adverse health outcomes including cancer [14,15], reproductive disorders [16], altered neurobehavior [17], cardiovascular disease [18], type 2 diabetes [19], and obesity [20]. In addition, BPA acts as an antiandrogen, affecting steps of the activation and function of the androgen receptor [21] and spermatogenesis in both animals [22] and humans [23].

Though not systematic, the present effort is a thorough review (173 references included in the full text), comprising in vitro, in vivo, and epidemiological studies that have assessed the effects of bisphenols (BPs), namely bisphenol A (BPA), its analogues, and its halogenated derivatives on the thyroid, considering their actions at different levels in cells or organs, in different animal models and in humans and their potential to exert a risk in causing a direct impact on the gland. Original studies published in English in peer-reviewed journals from 1 January 2000 to 31 October 2019 were searched in Pubmed through the search strategy: ((bisphenol* OR BPA OR tetrabromobisphenol* OR TBBP* OR tetrachlorobisphenol OR TCBPA) [ALL FIELDS]) AND (thyroid OR thyroid disorders OR thyroid function OR thyroid cancer OR thyroid nodule [ALL FIELDS]). From the initial search of 303 records, a total of 82 studies were selected by two authors on the basis of adherence to the purposes mentioned above. 

## 2. Bisphenols in the Environment and Humans

BPA is a monomer in the manufacture of polycarbonate plastics and epoxy resins widely used in diverse consumer products such as food and liquid containers, protective coatings inside metallic food and beverage cans and medical devices, as well as in flame retardants and thermal papers [24]. It is one of the 2000 endocrine disruptors known as “highest volume” chemicals, with an annual production of at least 8 million tons throughout the world [25]. 

BPA can be released from both effluent discharge of manufacturing plants and from transport, processing, and disposal of waste of BPA-containing products in landfills and incinerators [26]. Less than 1% of environmental BPA has been estimated to occur in the atmosphere, where it undergoes photo-oxidation and breakdown [27]. Nonetheless, the presence of BPA in the environment, though at low levels and despite the short half-life, is ubiquitous [28].

Due to its lipophilicity, detectable levels of the unconjugated form of BPA were measured in adipose tissue, brain, liver, and breast milk in humans (Table 1a). Moreover, BPA can pass through the placenta and amniotic fluid thereby exposing the fetus, as well as the developing infant, to exposure and accumulation [29] (Table 1a).

The first safety standard for humans set by the US-Environmental Protection Agency in 1988, adopted by the Food and Drug Administration as the reference dose and based on the LOAEL for BPA, was 50 micrograms per kilogram of body weight per day [30]. In 2013, the re-evaluation of BPA exposure and toxicity led the European Food Safety Authority to considerably reduce the safe level of BPA from 50 to 4 µg/kg/day (31). Human exposure to BPA is continuous and widespread, and diet is likely the major source of exposure in all population groups because of the ability of BPA to migrate from polycarbonate containers and metallic cans to food and beverages [31]. In 2011, the European Union banned the manufacture of baby bottles containing BPA [32], followed in 2012 by the Food and Drug Administration [33]. Infants and toddlers exhibit the highest estimated external average exposure because of their elevated consumption of food and beverages per kg of body weight [31] (Table 1a). Other routes of exposure are represented by inhalation of outdoor and indoor air, ingestion of domestic dust, dermal contact with thermal paper and cosmetics, and, for children, mouthing of toys [31]. The estimates for exposure to dietary and non-dietary sources are at least one order lower than the tolerable daily intake set by the European Food Safety Authority, except daily intake of infants fed with canned liquid formula in polycarbonate bottles (Table 1a).

BPA has a half-life in humans of about 6 h [34]. Following the oral exposure, in humans BPA is absorbed from the gastrointestinal tract and then metabolized in the liver, where is primarily conjugated with glucuronic acid to the non-active BPA-glucuronide, which is the main metabolite in urine and blood [34]. Urinary total BPA (conjugated + free), considered the most appropriate biomarker to assess human exposure [35], was detected among the different age classes in 88% to 98% of volunteers who participated in the National Health and Nutrition Examination Survey [35]. Significantly higher concentrations have been detected in children than in adolescents and adults whereas BPA levels measured in the blood of adults are approximately one order of magnitude lower than those found in the corresponding urine [36] (Table 1a).

Several structural analogues were introduced in the market to replace BPA [37]. Bisphenol F (BPF; 4,4′-dihydroxydiphenylmethane), bisphenol S (BPS; 4,4′-sulfonyldiphenol), and bisphenol Z (BPZ; 1,1-bis(4-hydroxyphenyl)-cyclohexane) are used in epoxy resin products [38], in cleaning products and thermal paper [39], and in highly heat resistant plastic materials and electrical insulation [40], respectively. BPAF (1,1,1,3,3,3-hexafluoro-2,2-bis(4-hydroxyphenyl)propane) is a fluorinated derivative widely used in the manufacturing of polycarbonate copolymers with 10,000 to 500,000 pounds annually produced in the United States [41]. BPA substitutes have been detected in various environmental matrices, and, with a few exceptions, their concentration values in urine are lower than those of BPA [42] (Table 1b). 

Tetrabromobisphenol A (TBBPA), a persistent compound synthesized by bromination of BPA initially replaced polybrominated diphenylthers, at present is the most widely employed brominated flame retardant, with a reported 2011 volume of 120 million pounds in the United States [43]. In the last years, tetrabromobisphenol S (TBBPS) and tetrachlorobisphenol A (TCBPA) have been extensively used as alternatives to TBBPA [44]. TBBPA is measured in the environment and in human body [45], and TBBPA exposure represents a significant health risk especially for children residing in an e-waste processing region [46] (Table 1c). 

## 3. Thyroid Disrupting Properties of BPs: in Vitro Studies

Biological function of thyroid hormone triiodothyronine (T3) is generally mediated by the nuclear receptors TRα1, TRβ1, and TRβ2 that are conserved in all vertebrates. T3 binds to the TRs with similar affinities mediating TH-regulated transcription with different levels in different tissues [60]. TRα1 is the predominant subtype in cardiac muscle and bone, TRβ1 is the predominant subtype in kidney and liver, while TRβ2 is more abundantly expressed in the hypothalamus and in the pituitary gland, and has a critical role in the regulation of the hypothalamic–pituitary–thyroid (HPT) axis [61]. TRs bind at DNA as homodimers or forms heterodimers with the retinoid X receptor to T3 response elements and they can regulate transcription both in the absence and in the presence of ligands [62]. On positively regulated genes, the unliganded TRs bind to corepressor proteins such as the silencing mediator of retinoid and thyroid hormone receptor (SMRT) or the nuclear receptor corepressor (N-CoR), resulting in the suppression of transcription [63]. The binding of T3 to TRs leads to a dissociation of the corepressors, and the subsequent recruitment of coactivator proteins, such as those of the p160/SRC (steroid receptor coactivator) family, including SRC1, SRC2, and SRC3, thus promoting activation of transcription [63].

In vitro models have tested and verified the ability of BPs to disturb thyroid function through multiple mechanisms that may produce different consequences depending on the heterogeneity of experimental conditions among studies such as the chemical tested, the concentrations used, and the presence/absence of T3 or T3 antagonists. BPs were reported to exert numerous effects on the thyroid, and each affected pathway may lead to perturbations of thyroid hormone levels, leading to a dysregulation of thyroid function. The pathways are not necessarily inter-connected, but there is some evidence that BPs may lead to an impact on the gland and its function at multiple levels, as reported in the following paragraphs.

### 3.1. Interference with T3 Transcriptional Activity

Numerous studies have evaluated the ability of BPs to suppress hormonal transcriptional activities mediated by TRα1 and TRβ1 in competitive binding and transient expression assays (Table 2).

**Figure 1:**
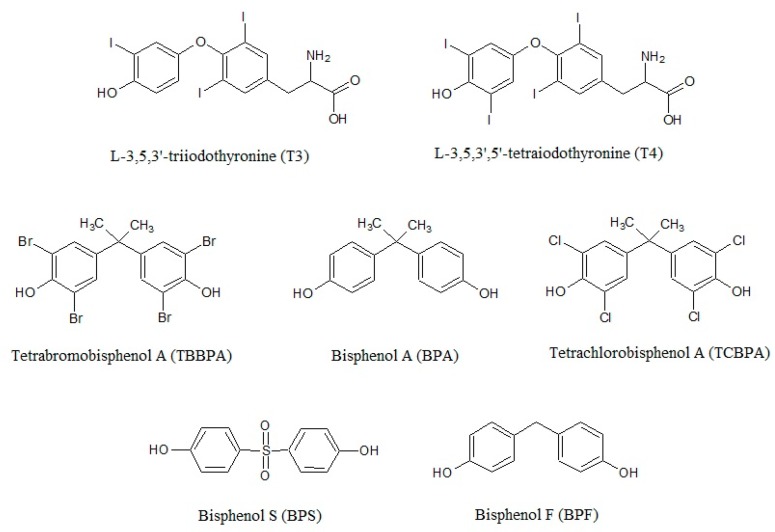
Bisphenol A, its analogues bisphenol F and bisphenol S, and the halogenated derivatives tetrabromobisphenol A and tetrachlorobisphenol A show a high degree of similarity with the thyroid hormones in regards the chemical structure.

BPs mainly acts as TR antagonists, inhibiting crucial processes related to development [64,65,66,67]. The TH signaling interference can occur by a direct binding of BPs to the receptor due to the high degree of structural similarity with THs (Figure 1) and preventing the binding of T3 [10,68,69,70,71]. Inhibitory effects of BPs on T3 hormonal activity were reported in different cell lines at doses of 10^6^–10^−4^ M [2,10,64,65,69,72,73,74], with brominated bisphenols showing a much stronger anti-TH activity than BPA and BPS [68].

Whereas BPA alone did not induce visible effects on T3-induced transcription [2,73,75,76], in the presence of physiological concentrations of T3, low-dose BPA enhanced the interaction of TR with N-CoR by directly binding to TR [2].

BPA may exert disrupting effects on TH-mediated transcription interfering with a different non-genomic mechanism mediated by integrin αvβ3, a heterodimeric transmembrane glycoprotein [77]. In normal conditions, T3 and thyroxine (T4) induce serine phosphorylation of TR-β1 by binding to αvβ3 and activating mitogen-activated protein kinases (MAPK) and/or c-Src/PI3K pathways [78], which determines the dissociation of N-CoR or SMRT from TR-β1 and consequent activation of transcription. The competitive binding of BPA to αvβ3 antagonizes the serine phosphorylation of TR-β1 leading to the recruitment of N-CoR/SMRT to TR-β1 and suppression of transcription [79].

A few studies observed that, in the absence of T3, BPs behave as TR agonists [65,69,70,72,80] and this thyromimetic effect can occur at very low concentrations (10^−8^–10^−7^ M) [69,70] and disappear at high doses (10^−4^ M) [65,69], showing a biphasic concentration-response relationship.

### 3.2. Cell Proliferation

The rat tumor pituitary cell line GH3 has been frequently employed as a standard pituitary cell model for assessing TH effects [81]. Indeed, cell proliferation and growth hormone (GH) secretion primarily depend on THs [81] and involve TR-mediated mechanisms, specifically the induction of gene expression [82].

A series of investigations assessed the agonistic and antagonistic properties of BPs in GH3 cell growth both in absence and in presence of T3 (Table 2). BPA, and in particular BPA derivatives, generally promoted GH3 cell proliferation and GH release in the concentration range of 10^−6^–10^−4^ M [70,81,83]. In some studies, the agonistic activity was detected exclusively in the presence of T3 [82,84], whereas in others BPA and its substitutes inhibited cell growth with T3, and TH-antagonistic effects appeared to depend on the tested dose and the time of exposure [80,85].

Effects of BPs on cell growth were antagonized by amiodarone, a known TR antagonist [80]. Nonetheless, amiodarone was also reported to act as a slight agonist at low concentrations and antagonist at increasing doses, and BPA and its halogenated derivatives exhibited comparable dose–response curves [76]. In PTC cells, BPA had similar proliferative effects as E_2_ [86], and consistent with this finding, co-exposure to E_2_ potentiated the increased GH3 cell proliferation (from 190% to 252% after 96 h) by BPA and BPAF [85]. In contrast, TBBPA could not counteract the inhibitory effect of fulvestrant, a strong antiestrogen, on cell growth [81].

Cell growth was further antagonized by U0126, an inhibitor of MEK, the kinase responsible for the activation of ERK in the Raf–MEK–ERK pathway in mammalian cells [87]. Similarly, TBBPA at concentrations in the lower micromolar range caused arrest of cells growth in the G1 or G2 phase, depending on the duration and intensity of the treatment and on cell specific and dose dependent modulations of the Raf–MEK–ERK pathway [87].

### 3.3. Cytotoxicity

MAPKs have an important role in cellular signaling pathways, and the kinases JNKs/SAPKs and p38 MAPKs are often activated by cellular stresses and thus primarily linked to cytokine biosynthesis and induction of apoptosis [88]. Thus, any interference of exogenous chemicals with kinases and phosphatases involved in cellular signaling processes can result in possible cytotoxic effects, including cell death [87].

Similar to cell proliferation, cell viability has been evaluated in cell lines exposed to BPs (Table 2).

Cytotoxicity was observed after exposure to BPA and its halogenated derivatives at a concentration range of 10^−5^–10^−4^ M; alone and/or with T3 [10,73,76,82,87]. TBBPA was found to produce cytotoxicity 100 times higher than BPA [75] although in other cell models comparable doses of BPA, TBBPA, and TCBPA did not cause changes in cell viability [65,70,89].

### 3.4. Competitive Binding with Thyroid Hormone Binding Proteins

One of the possible mechanisms of BPs for disrupting TH homeostasis is the competitive binding with serum transport proteins due to the structural similarity to T4 and T3. THs mainly bind to three transport proteins in human serum, namely thyroxine-binding globulin (TBG), which is responsible for 75% of the specific T4 binding activity, transthyretin (TTR), and human serum albumin [90]. A few studies tested the capability of these chemicals to compete with THs for binding to TTR, which in non-mammalian vertebrates exhibit a higher affinity for T3 than T4, whereas in human plasma is responsible for only 10% to 15% of the TH transport [91] (Table 2).

Meertz et al. [92] found no TTR binding for 17 polybrominated diphenyl ethers at maximum concentrations confirming that hydroxylation at the *para* position with at least one adjacent halogen substituent could represent a prerequisite for TTR binding. Indeed, TBBPA was the most potent competitor among the phenolic compounds tested, binding to TTR in a range from 1.6 [84] to 10.6-fold [92] stronger than the natural ligand T4. Moreover, the affinity of TBBPA for TTR was three times greater than that of BPA [70], and this is in line with the higher binding affinity of halogenated derivatives for TRs compared with BPA [10,71]. Actually, the hydroxylated derivatives of BPA also exhibited a strong affinity for TBG, as elucidated in a transport protein-based biosensor assay [93]. Using a fluorescent probe, Cao et al. observed that BPA affinity for TTR and TBG was weaker than T4 by 300 to 2666 fold; hence the current levels of BPA in humans are unable to interfere with T4 serum transport [90].

### 3.5. Perturbation of Thyroid Hormone Uptake

Thyroid hormone uptake into target cells is controlled by membrane bound transporters, such as monocarboxylate transporter (MCT) 8, MCT10, and multiple members of the Na-independent organic anion transport protein (OATP) family [94]. OATP1C1, in particular, shows a high degree of tissue selectivity, being expressed predominantly in brain and testis, and high preference for T4 and reverse T3 as the ligand [95], and it facilitates the transport of T4 across the blood–brain barrier [96]. In different species, MCT expression has been detected in numerous tissues including the brain wherein MCT8 is responsible for the neuronal uptake of T3 [95]. Mutations in the *MCT8* gene cause a severe X-linked psychomotor retardation associated with highly elevated serum T3 levels and decreased T4 concentrations whereas the thyroid-stimulating hormone (TSH) values remain in the normal or slightly elevated range levels [97].

In a recent study performed in cells overexpressing the human MCT8 gene, among the several common environmental contaminants classified as flame retardants, pesticides, plasticizers, and others that are suspected to disrupt TH signaling, only BPA was observed to reduce T3 uptakes to around 60% and 40% of the control at concentrations (125 μM) below those that reduced cell viability <80% [94]. This finding is consistent with an earlier study that detected a slight inhibition of T3 transport capabilities of MCT8 by BPA, though at concentrations likely higher than those occurring in vivo [98].

### 3.6. Dysregulation of Gene Expression

In addition to the ability to interfere with TR signaling throughout a direct binding to the receptor, a number of studies observed that BPs may directly affect thyroid gene expression (Table 2).

At doses as low as 10^−6^ M, BPA and its analogues induced expression of transcripts of genes implicated in thyroid cell activity and proliferation (e.g., the *Thyroid stimulating hormone-receptor* (*Tsh-r*)), TH biosynthesis (e.g., *Tg, Sodium iodide symporter* (*Slc5a5* encoding NIS), *Thyroid-peroxidase* (*Tpo*) and their transcription regulators (e.g., *Paired box 8* (*Pax8*), *NK2 homeobox 1* (*Nkx2-1*), and *Forkhead box E1* (*Foxe1*)) by over 1.5 fold [89,99,100]. Conversely, BPA did not markedly affect transcriptional expression of *Slc5a5, Nkx2-1,* and *Tpo* but inhibited NIS-mediated iodide uptake [100]. BPA increased the expression of *Tg* gene in the presence of increasing TSH amounts, suggesting a potency similar to that of TSH in enhancing *Tg-*promoter activity [94]. The authors also reported that two anti-estrogens, which alone induced the activity of the *Tg* promoter, were not able to enhance BPA activity on the *Tg* promoter, indicating that the effects triggered by BPA do not necessarily involve ER signaling [89]. BPA at the nanomolar range significantly impaired the transcriptome of thyroid cells in a time dependent manner [101]. In fact, whereas short-term exposure to BPA did not cause any relevant transcriptomic changes, long-term exposure, though unable to exert visible damage on cells, determined a slight deregulation of many genes involved in cell proliferation/death, cancer, and DNA repair [101].

BPA and its analogues BPZ, BPF, and bisphenol M (BPM; 4,4′-(1,3-phenylenediisopropylidene)bisphenol) suppressed the transcription of several genes involved in the regulation of the HPT axis (e.g., *TSH-specific β subunit* (*Tshβ*)*, Thyroid hormone receptors* (*Trα* and *Trβ*)*,* and *Deiodinases* (*Dio1* and *Dio2*)) in a concentration range of 10^−7^–10^−6^ M, with BPA substitutes being able to disrupt thyroid regulation at lower doses than BPA [75,85,99]. Furthermore, co-exposure with E_2_ potentiated decreased expression of *Trα, Trβ* and *Dio2* [85].

BPA inhibited the activities of DIO1 and DIO2 [102], and both BPA and TBBPA markedly dysregulated transcription of *Dio3*, which is responsible for protection of tissues from TH excess and is the predominant deiodinase expressed in human placenta [103], and hepatic phase II metabolizing genes (*Sulfotransferases* (*Sult1*) and *UDP*-*glucuronosyltransferases* (*Ugt*)) [75]. TBBPA, but not BPA, increased expression of the *Ttr* gene [75], an action at mRNA level that corroborates the competing binding capabilities of TBBPA with TTR [70,84].

## 4. Thyroid Disrupting Properties of BPs: in Vivo Studies

In vivo effects of BP exposure on thyroid function/action are contradictory and difficult to compare, as a consequence of the scarce number of studies performed especially in mammals, the different models, and diversity of the experimental conditions, i.e., the chemical used, the time and dose of treatments, the outcomes assessed. In regards the risk of thyroid cancer associated to BPs exposure, the subject remains almost entirely unexplored.

### 4.1. Rodents

In rodents, most of the studies have been performed in pregnant females, and have evaluated the variations of TH levels in mothers and pups following prenatal and/or lactational exposures (Table 3).

In accordance with numerous in vitro studies, BPA can act as a selective TH antagonist on TRβ, inhibiting TH-negative feedback. Indeed, Zoeller et al. [104] and Zhang et al. [105] observed a significant increase of serum T4 levels in pups of both sexes and in female adults, respectively, without any apparent interference on TSH release. In male adult rats, treatment with BPA led to an increase of T4 levels and a reduction of the T3/T4 ratio, suggesting that in exposed animals BPA may impair the peripheral conversion of T4 to T3 [102].

In other experiments, BPA exposure did not produce significant variations in plasma T4 levels [106,107,108,109] or, alternatively, the effects may not endure after BPA removal and metabolism [110,111,112]. It is still unclear whether exposure to BPA and its derivatives can cause hypothyroidism due to limited evidence. A decrease in T4 levels was found in male and female adult rodents [110,113,114] and in rat pups of both sexes [114] or with a sex-specific effect [112,115]. The competition of BPs with TTR, as observed in vitro [70,84,92], resulting in a portion of serum T4 displaced from TTR, could determine an increased rate of T4 metabolism and elimination and the consequent reduction of T4 circulating levels [110].

Perinatal or neonatal exposure to BPA was associated with a significant increase of TSH levels in juvenile males [116] and in females in estrus [117,118], accompanied by a significant increase of GH levels and an impaired sensitivity of the thyroid gland to TSH stimulation, respectively, both of which indicate an alteration of the HPT axis [117]. On the other hand, the reduction of serum T3 or T4 after TBBPA treatment induced feedback stimulation, as suggested by the increased pituitary weight [114], whereas in other studies this was insufficient to affect serum TSH or TH levels, thyroid histopathology, and thyroid weight [107,110].

Adult males treated with BPA showed a decrease activity of hepatic DIO1, coherently with what has been reported in vitro [102]. Moreover, in female adult rats BPA lowered thyroid iodide uptake and thyroid peroxidase (TPO) activity, which are two essential steps in TH biosynthesis, probably due to an elevation of reactive oxygen species (ROS) production. Both NIS and TPO have been found to be sensitive to ROS [119,120], and in particular the decrease in TPO activity could be attributable to the oxidation of this enzyme [106]. Increased expression of pituitary *Tshβ* was reported in female rat neonates exposed to BPA [117], whereas Silva et al. did not find any significant reduction of *Tshβ* mRNA levels in treated female rats [106].

To date, it remains unclear whether BPA plays a role in the pathogenesis of thyroid carcinoma. Zhang et al. recently demonstrated that BPA could enhance the susceptibility to TC [105]. Rats pre-treated with N-bis (2-hydroxypropyl) nitrosamine, a drug stimulating thyroid proliferation and promoting a cancerous phenotype [121] and then exposed to BPA and excess iodine for 64 weeks, exhibited a significant increase in incidence of TC and thyroid hyperplasia lesions as well as the up-regulation of ERα in the hyperplasia lesions. The authors speculated that BPA could increase ERα expression in the thyroid, which possibly participated in the proliferation process [105].

### 4.2. Sheep

Sheep are considered a more relevant model to humans than rodents to evaluate fetal exposure to thyroid disruptors and their effects on the mother/newborn thyroid functions because of a similarity in the timing of the ontogenesis of thyroid [122]. In both species thyroxine binding globulin is the main blood transport protein for THs [123], and thyroid system maturation is qualitatively similar in the sheep and human fetuses, although the total maturation time is different (165 days vs. 300 days) [122,124].

Two studies have investigated the relationship of BPA exposure with thyroid function (Table 3). Viguié et al. [122] reported that BPA exposure of pregnant ewes was associated with a transitory hypothyroxinemia of both mothers and their newborn lambs, with a significant reduction of both circulating total T4 (TT4) and free T4 (FT4), findings in agreement with rodent studies [111,112]. In a following study, the authors confirmed alterations of gestational thyroid function, observing a significant reduction of FT4 and total T3 (TT3), but not TT4, in pregnant ewes treated with environmentally-relevant BPA concentrations via subcutaneous and dietary routes of administration [123]. After subcutaneous administration, the maximum serum concentration of BPA obtained was significantly higher (0.4 nmol/mL vs. 0.1 nmol/mL) and more prolonged than after dietary administration [123].

### 4.3. Zebrafish

Numerous studies have been published on the use of zebrafish (*Danio rerio*) to explore the effects of EDCs on the thyroid, due to several advantages: a short life cycle, high rates of production, real-time observations during the entire embryonic development, and high conservation of the molecular mechanisms regulating thyroid development with those of mammals [125,126]. The early life of fish, in particular, is acknowledged as highly sensitive to the effects of EDCs [127].

Coherently with results observed in vitro and in rodents, BPs may disturb TH homeostasis and gene expression in zebrafish embryos/larvae (Table 3).

Positive [37,38,128] and negative [37,38,128,129,130,131] associations between exposure to BPs and T3 and/or T4 levels have been reported, depending on the chemical used, the dose tested, and the time of exposure. Reductions in T4 concentrations, when accompanied with higher TSH contents, may compensate hypothyroidism in zebrafish larvae and stimulate TH synthesis [38,129]. Some experiments observed an interaction between TH levels and sex [129,131]. Tang et al. showed a reduction in whole-body TT4 and TT3 levels but not a significant variation of ratio TT3/TT4, which indicates the relative normal TH homeostasis [130].

Similarly, BPs disrupting effects on thyroid gene expression vary according to the different experimental conditions, especially the duration of exposure [126]. Hence, transcription levels of genes implicated in thyroid cell function and proliferation (*Tsh-*r), TH activity (*Trα, Trβ*), and transport (*Ttr*) can be up- [37,68,126,130,132,133] or down-regulated [38,68,126,128,130,133]. The transcription of *Hematopoietically expressed homeobox* (*Hhex*) was up-regulated in larval fish following exposure to BPA or BPF, although it is important to note that the *Hhex* gene is expressed in early life, contributing to differentiation and development of the thyroid gland, as well as of other organs, such as the pancreas and liver [37].

Increased [89,128,129] or decreased [130,132,134,135] expression of *Slc5a5, Tpo, Pax8,* and *Tg* transcripts was dependent on the dose and window of exposure. Additionally, BPA and BPS appeared to interact with PAX8 and thyroid transcription factor 1 (TTF1) in silico [135]. Genes such as *Tpo, Tg,* and *Slc5a5* have binding sites for PAX8 or TTF1 on their enhancer or promoting regions. Differences of interactions between BPs and the transcription factors could be attributable to stimulation or inhibition with varying BPs doses, and produced as final effect altered expression of the genes controlled by PAX and TTF1 [135].

BPs stimulated thyroid signaling increases expression of *Corticotrophin-releasing hormone* (*Crh*) mRNA in the hypothalamus [37,38,129] and of *Tsh* and *Tshβ* in the pituitary gland [89,128,130,132,133,134], except for TBBPA, which down-regulated *Tshβ* mRNA in embryos [133].

Exposure to BPA and BPA analogues further induced transcription of genes involved in TH metabolism, i.e., *Dio1* and/or *Dio2* [37,129,132,134], which are implicated in activation/inactivation of T4 and in conversion of T4 to T3 in peripheral tissues, respectively [136,137], and *Ugt1ab* [37,38,129].

Notably, co-treatment with T3 appeared to reverse or eliminate thyroid disrupting effects of TBBPA on THs levels and gene transcription in zebrafish larvae [128], whereas a combined exposure of BPAF and sulfamethoxazole, an antibiotic used especially in aquaculture, produced more pronounced changes in transcription levels [134].

## 5. Thyroid Disrupting Properties of BPs: Human Studies

Perturbations in THs parameters consequent to exposure to BPA have been documented in humans, i.e., the general population, pregnant women, or occupational settings, although the study design, predominantly cross-sectional, does not allow establishment of any causal relationship. Research has highlighted positive, negative, or null associations with T4 levels, whereas a few prospective birth cohort studies suggest that prenatal BPA exposure may modify THs normal serum concentration in a sex-specific manner. Several investigations have demonstrated BPA-induced disruption of thyroid function by altering serum TSH levels. This effect could occur from a direct action of BPA on the pituitary gland via the estrogen signaling pathway or, alternatively, from a transient increase of T3 or T4 production that could lead to a feedback mechanism and the subsequent release inhibition of TSH. Overall, discrepant results among studies may be attributable to BPA levels, time of exposure, iodine intake, differences in age, ethnicity, diet, socioeconomic status, and the determination methods of THs, while at present the potential role of BPs in thyroid carcinogenesis in humans remains to be deeply explored (Table 4). It is noteworthy that the absence of adjustment for other confounding factors such as co-exposure to other EDCs makes the overall evaluation of thyroid dysfunction related to BPs exposure complex. Furthermore, a comparison between effects observed in animal models with those reported in epidemiological studies is complex given different serum T4 half-lives (12–24 h in rats vs. 5–9 days in humans), metabolic pathways of BPs, and doses of exposure, which in humans is more likely to be chronic and low level [138].

### 5.1. Effects on Serum TH Levels

Studies carried out in the general population and in mother/child cohorts showed positive [139,140,141], negative [142,143,144,145], and no associations between BPA exposure and serum T4 levels [143,146,147,148,149,150,151], whereas the relationship with serum T3 levels was estimated in few studies, with different findings [139,141,142,149,150].

In agreement with results from studies in animals [104,105,108], exposure to BPA led to TSH release/suppression independent of alterations in circulating THs levels [143,145,147,148,150,152] or, less frequently, was associated with variations of serum T4 levels [139,149].

There was no association of BPA with hypothyroidism in Japanese women with a history of recurrent miscarriages [151], nor any significant relationship between serum TBBPA in Korean infants with congenital hypothyroidism and THs levels [140]. Conversely, middle-aged and elderly Chinese with overt or subclinical hyperthyroidism had higher urinary BPA than euthyroid subjects [151], and an increased content of urinary BPA was also observed in obese adults undergoing a diet program or bariatric surgery compared to lean controls, probably due to differences in food intake [152].

Sex-related differences in the relationship between BPA and THs were reported both in the general population [142] and in newborns [143,148], coherently with studies performed in rats [112,115], and possibly attributable to a less efficient ability to metabolize BPA, i.e., a reduced expression of *uridinediphosphate-glucuronosyltransferase 2B1* in male compared to female livers [153], or to a different androgen-related metabolism of BPA [154].

The interactions between BPA and THs during pregnancy and fetal development have been recently studied. The association between BPA and TSH levels in newborns was stronger when the time elapsed between the two measurements was shorter [143,148], suggesting that specific windows of exposure may influence susceptibility to BPA or, alternatively, that a transient effect on the HPT axis may occur, as shown in rodents [111,112]. However, the inverse association of BPA–TSH in pregnant women detected through repeated measures as well as stratified analyses by visit could indicate the absence of a specific window of vulnerability [139].

### 5.2. Association with Thyroid Diseases

The influence of BPA on thyroid autoimmunity is controversial (Table 4). Whereas urinary BPA concentration was associated with variations of TH levels both in children and adults of both sexes, independent of serum thyroglobulin antibodies (TgAb) and thyroid peroxidase antibodies (TPOAb) [149,155], another study found a positive relationship between serum BPA and TPOAb in men and women [156]. Moreover, a significant negative correlation of serum BPA with FT4 in male subjects was found only after exclusion of subjects with positive thyroid antibodies, suggesting that TgAb might be a mediator of the relationship between BPA and FT4 [144].

Kim and Oh reported a slight positive correlation between serum TBBPA and thyroid-stimulating hormone receptor antibodies, indicative of metabolic diseases, in mothers of infants with congenital hypothyroidism, suggesting that brominated derivatives of BPA might affect thyroid function status [140].

Recent investigations have explored the role of BPs as risk factors of occurrence of thyroid nodules (TNs), palpably and/or ultrasonographically discrete lesions, distinct from the surrounding parenchyma of the thyroid gland, which are either benign or malignant [157]. A study reported no association between BPs and higher risk of TNs in adult females [147], whereas Wang et al. observed an inverse correlation of urinary BPA and the risk of multiple TNs but not of solitary TNs in schoolchildren [158]. On the other hand, a significant near linear association between BPA and higher risk of TNs was observed exclusively among participants positive for TgAb and TPOAb [159]. Both urinary BPA and creatinine-adjusted BPA levels were higher in Chinese women with TNs than those without TNs [159], which is consistent with the increased urinary BPA contents in patients with nodular goiter and PTC (160), while median urinary BPA levels were lower in the cases compared to controls among women from Cyprus and Romania [147]. In the study by Zhou et al. [160], which was aimed to investigate the relationship between BPA and iodine exposure with nodular goiter and PTC, sex-specific associations were shown, with higher concentrations of BPA in women than in men affected by PTC and nodular goiter, and a lower urinary BPA content in the female PTC group than the female nodular goiter group, probably due to differences in BPA elimination rates. Marotta et al. recently found a significant dose-independent correlation between BPAF and the risk of differentiated TC in subjects with TNs. Of note, this association was not related to an increase of TSH levels, indicating a potential direct mutagenic action of BPAF on thyroid cells [161].

## 6. Discussion

The thyroid is highly susceptible to environmental pollutants, which may act as either genotoxic or non-genotoxic carcinogens [147]. BPA is a widespread chemical detected in urine specimens of the majority of adult populations. BPA analogues and derivatives are ubiquitous contaminants, measured in environmental and biological matrices, exhibiting a thyroid disrupting potential comparable and even stronger than BPA. The mechanisms of BPs action on THs are complex and need to be still elucidated.

Overall, the in vitro studies demonstrate that BPs may bind to TRs, acting mainly as TR antagonists, but also as agonists or without exerting any effect on TH signaling. Similarly, different patterns of *Trβ* expression following BPs exposure were observed in in vivo models. THs and their receptors regulate many important processes such as proliferation, differentiation, and apoptosis, and since TRβ is the major isoform in the thyroid, it can be hypothesized that disruption of its expression, leading to abnormalities in T3-induced transcriptional activity, could be involved in tumorigenesis [72,162].

In vivo experiments, supported by in vitro evidence, highlighted the ability of BPA and its substituting chemicals to affect thyroid follicular cell gene expression, particularly transcriptional levels of those genes encoding for factors involved in THs synthesis (TPO, NIS, Tg, PAX8). Up-regulation of *Tg* and *Slc5a5* transcript levels may promote thyroid development to compensate for the depressed T4 concentration, as also reported for polybrominated diphenyl ethers [163]. Transcriptional levels of deiodinases were more elevated in exposed zebrafish, in accordance with a study reporting that hypothyroidism caused by EDCs is associated with higher activity and expression of *Dio2* [164]. On the other hand, a recent study reported a reduction of liver DIO1 activity in BPA-treated adult rats [102], which is a finding worth of note as decreased expression of DIO1 was observed in nearly all PTCs and is likely an early event in malignant TC [165].

In rodents and in two different types of cell lines, BPA up-regulated *Pax8* transcripts, suggesting a role of BPA in increasing *Pax8* expression independent from the cellular context [89]. PAX8 is a cell-lineage-specific transcription factor that has been mainly characterized in the thyroid gland for its role in thyrocyte differentiation, and it has been revealed as a potential diagnostic marker for several cancer sites including TC [166].

TSH should represent an effective index of activation of the HPT axis to evaluate central effects of xenobiotics on thyroid function through measurement of TSH secretion or expression as a compensatory mechanism for maintaining TH homeostasis. Moreover, TSH levels are an independent predictor of thyroid nodule malignancy regardless of age, sex or family history [6]. Increased expression of TSH and TSHβ observed in vivo was also reported after exposure to pesticides and halogenated chemicals in fish [163,167] suggesting that elevated production of TSH could represent one of the mechanisms of action of BPs, as already hypothesized for other EDCs (9). In pituitary cells, BPA and E_2_ could further induce release of TSH desensitizing the response to thyrotropin releasing hormone from hypothalamus [118]. In contrast, humans and pregnant ewes exhibited hypothyroxinemia after BPA exposure without significant modifications of TSH, whilst other epidemiological studies reported a decreased TSH production, probably consequent of a direct action of BPA on pituitary gland through estrogen receptor signaling or of a feedback mechanism triggered by BPA-mediated perturbations on circulating T3 and T4 [139].

The frequency of chronic autoimmune Hashimoto’s thyroiditis, the most common cause of primary hypothyroidism in western countries, has increased in the last two decades, and a variety of factors such as tobacco smoking, iodine and selenium intake, and exposure to EDCs, may contribute to the elevated incidence by interacting with susceptibility genes (6). Autoimmune thyroiditis may coexist with TC [168], and a recent meta-analysis demonstrated that this condition predisposes patients to the development of the papillary histotype [169]. Thyroid autoantibodies were reported to be positively associated with the level of urinary BPA, therefore subjects with thyroid autoantibodies positivity, as characterized by immune dysfunction and a lower ability to eliminate damaged cells, are probably more vulnerable to the effects of BPs on TNs [159].

It cannot be excluded that exposure of thyrocytes to BPA involves hydrogen peroxide generation due to an elevated activity of a calcium-dependent NAPDH oxidase (DUOX) [106]. TPO is a key enzyme in the synthesis of THs, catalyzing, through the cofactor H_2_O_2_, the iodination of tyrosyl residues in Tg [106]. Thus, the increased oxidative stress in the thyroid gland, which is related to a reduction of TPO activity, corroborates the negative correlation between TPO and DUOX2 in thyroid nodular lesions [170]. Furthermore, oxidant/antioxidant balance was recently reported to be impaired in children affected by autoimmune thyroiditis, though it is unclear whether oxidative stress is the real cause of the disease or the likely consequence of exposure to EDCs, including BPA [155].

Finally, BPA is potentially linked to excess iodine in the pathogenesis of the nodular goiter and TC in animals [105] and humans [160]. High urinary iodine is a risk factor for the development of benign TNs and PTC [171], being associated with reduced expression of NIS, an early abnormality in the pathway of thyroid cell transformation, and increased occurrence of *BRAF* mutations [172], which are both hallmarks of differentiated TC [173].

## 7. Conclusions

This review aims at evaluating the extensive body of experimental and human studies that in the last two decades have attempted to explore the effects of BPA, its substitutes, and its halogenated derivatives on the thyroid at different levels. Despite the variety of approaches applied and the heterogeneous and sometimes even conflicting results from the examined studies, a series of interesting indications supports the hypothesis of a role of BPs in interfering with the normal thyroid function. Although the toxicity pathways of BPs on the thyroid need to be further elucidated, BPA analogues and halogenated derivatives do not emerge as safer alternatives to BPA in term of TH disruption. There is evidence that BPs alters THs circulating levels, inhibiting TH-negative feedback, act as selective TR antagonists, and interfere with expression of genes involved in thyroid stimulation, TH synthesis, TH activity, and TH transport and metabolism. Several reported findings, mainly from experimental studies, are, however, rather inconsistent, while the association of BPs exposure with thyroid cancer is so far almost unexplored. The lack of uniformity in experimental methodology, as well as substantial differences in populations investigated in epidemiological studies, do not allow definitive conclusions to be drawn. Standardized in vivo, in vitro, and in silico studies are recommended to evaluate the physiopathology of the damage associated with exposure to environmentally relevant levels of BPs, identify other potential molecular targets, and clarify the structure−activity relationship of BPs. At the same time, large population-based human studies with prospective designs and repeated measures of urine BPs concentrations and thyroid volume over time, as well as an accurate control of confounders, should be performed for the assessment of the temporal relationship between markers of exposure and long-term effects.

## Figures and Tables

**Table 1:** Concentration of bisphenols in the environment and human body, and estimated exposure by age groups to bisphenol A (a), principal bisphenol A substitutes (b), and halogenated derivatives of bisphenol A (c).

**(a) Bisphenol A**		
**Environmental Matrix**	**Concentration**	**Reference**
Surface water	nd-1.95 µg/L	[47]
Sediments (industrialized areas)	nd-13,370 µg/kg dry weight	[41]
Soil	<0.01–1000 µg/kg	[29]
Indoor dust	nd-39.1 µg/g	[48]
Atmosphere	10^−3^–1.74 ng/m^3^	[49]
Landfill leachate (hazardous waste site)	Up to 17,200 µg/L	[50]
**Human Body**	**Concentration**	**Reference**
Brain	Mean: 0.91 ng/g	[51]
Liver	Mean: 1.30 ng/g	[5]
Adipose tissue	Mean: 3.78 ng/g	[51]
Breast milk	Mean: 0.61 µg/L	[52]
Blood (adults)	Mean: 0.20 µg/L	[36]
Cord blood	Mean: 0.13 µg/L	[36]
Urine (European adult population)	Geometric mean: 2.5–3.6 µg/L	[31]
Urine (North America children)	Geometric mean: 1.3–3.7 µg/L	[31]
Urine (North America adults)	Geometric mean: 1.0–2.6 µg/L	[31]
**Age Group/Source of Exposure**	**Average External Exposure**	**Reference**
Infants (0–3 month)/Formula fed from polycarbonate bottles	2.4 µg/kg/day	[53]
Infants (0–6 month)/Formula fed from non- polycarbonate bottles	0.03 µg/kg/day	[31]
Infants (6–12 month) and toddlers (12–36 month)/Diet	0.375 µg/kg/day	[31]
Infants (6–12 month) and toddlers (12–36 month)/Oral dust and toys	0.007–0.009 µg/kg/day	[31]
Infants (0–12 month) and toddlers (12–36 month)/Inhalation	0.7 µg/kg/day	[31]
General population (>3 years)/Diet	0.116–0.290 μg/kg/day	[31]
General population (>3 years)/Thermal paper	0.059–0.094 µg/kg/day	[31]
General population (>3 years)/Cosmetics	0.002 µg/kg/day	[31]
General population (>3 years)/Inhalation	0.2–0.4 µg/kg/day	[31]
**(b) Principal Bisphenol A Substitutes**		
**Environmental Matrix**	**Concentration**	**Reference**
Surface water (BPF)	nd-2.850 µg/L	[47]
Sediments in industrialized areas (BPF)	nd-9650 µg/kg dry weight	[41]
Indoor dust (sum of several bisphenols including BPF, BPS, BPZ)	0.00083–26.6 μg/g	[49]
**Human Body**	**Concentration**	**Reference**
Urine (BPS: general population—USA/Asian countries)	Geometric mean: 0.030–1.18 µg/L	[54]
**Age Group**	**Estimated Exposure**	**Reference**
Children and adolescents (<20 years)—USA/Asian countries (BPS)	Median: 0.009 μg/kg/day	[54]
Adults (≥20 years) – USA/Asian countries (BPS)	Median: 0.004 μg/kg/day	[54]
**(c) Tetrabromobisphenol A**		
**Environmental Matrix**	**Concentration**	**Reference**
Atmosphere (e-waste dismantling site)	66.01–95.04 ng/m^3^	[55]
Indoor dust	42.21–46,191 ng/g dry weight	[46]
Sediments	Up to 518 ng/g	[56]
Soil (industrialized areas)	1.64–7758 ng/g dry weight	[57]
Surface water	0.85–4.87 μg/L	[56]
**Human Body**	**Concentration**	**Reference**
Breast milk	4.110 ng/g lipid weight	[58]
Cord serum	Mean: 0.199 ng/g fresh weight	[58]
**Age group/Source of Exposure**	**Average External Exposure**	**Reference**
Infants/Breast-feeding	<0.00018–0.171 μg/kg/day	[59]
Infants/Dust ingestion (e-waste recycling site)	0.00031–0.054 μg/kg/day	[46]
Adults/Dust ingestion (e-waste recycling site)	0.00004–0.0075 μg/kg/day	[46]
Adults/High fish consumers	0.00026 μg/kg/day	[59]

**Table 2:** Summary of in vitro studies analyzing effects of bisphenols on thyroid hormoneresponsive cell lines.

Species	Model	Method	Exposure Time	Doses Tested	Principal Results	Reference
***Interference with T3 transcriptional Activity***						
Yeast *(Saccharomyces* *cerevisiae* Y190)	Yeast cells	Recombinant two-hybrid yeast assay	2.5 h	0.005 nM–50 μM BPA/BPS/TBBPA/TBBPS ±10^−4^ M T3	Antagonistic activity of BPs toward TRβ in a dose-dependent manner, with TBBPS showing the strongest antagonistic activity. IC_10_ BPA = 884 ± 65.3 nM IC_10_ BPS = 312 ± 25.9 nM IC_10_ TBBPA = 21.1 ± 9.6 nM IC_10_ TBBPS = 10.1 ± 5.1 nM	[68]
Yeast *(Saccharomyces* *cerevisiae* Y190)	Yeast cells	Yeast two-hybrid assay	4 h	10^−8^–10^−4^ M BPA/TCBPA/TBBPA ± rat liver S9 preparation	Agonistic activity of TBBPA toward TRα with a dose-dependent response curve. After exposure to S9 metabolic activation, increase of agonistic activity of TBBPA and TCBPA. No activity of BPA.	[69]
				0/1.6*10^−7^/8*10^−7^/4*10^−6^/2*10^−5^ M BPA/TCBPA/TBBPA ±100 nM T3 ± rat liver S9 preparation	With T3, significant antagonistic activity of TBBPA and TCBPA toward TRα, enhanced by exposure to S9 metabolic activation at the same concentration.	
Zebrafish (*Danio rerio*)	Hepatocyte (ZFL cells)	Luciferase reporter gene assay	24 h	5/10/25/37.5% LC50 BPA/TBBPA	No induction of TR transcriptional activity by BPA or TBBPA alone.	[75]
				1/2.5/5/10/25% LC50 BPA/TBBPA +0.1 nM T3	With T3, decrease of transcriptional activity by BPA.	
Amphibian (*Xenopus laevis)*	Tadpole tail culture	Semi-quantitative RT-PCR	5 days	10^−7^–10^−5^ M BPA	Inhibition of *Trα* and *Trβ* mRNA expression with a greater effect on the expression of *Trβ* than *Trα*. Moderate suppression of *RXRγ* mRNA expression.	[66]
				10^−5^ M ±2.5*10^−8^/10^−7^/4*10^−7^ T3	T3 counteracts the inhibitory effects of BPA on *Trα* and *Trβ* mRNA expression, and, in the case of *Trβ,* dose-dependently. Dose-dependent suppressive effect of T3 on *RXRγ* gene expression independently of the presence of BPA.	
Amphibian (*Xenopus* *laevis)*	XL58-TRE-Luc cells	Luciferase reporter gene assay	24 h	10^−8^–10^−6^ M BPA/TBBPA ±2 nM T3	With T3, inhibition of transcription in a dose-dependent manner. In the absence of T3, agonistic activity.	[70]
Amphibian (*Xenopus laevis)*	XL58-TRE-Luc cells	Luciferase reporter gene assay	24 h	0/10^−8^–10^−6^ TBBPA ±2 nM T3	With T3, inhibition of transcription in a dose-dependent manner.	[74]
Chinese hamster	Ovary (CHO-K1 cells)	Luciferase reporter gene assay	24 h	10^−10^–10^−4^ M BPA/TBBPA/TCBPA	Suppression of transcription in cell transfected with TRα1 or TRβ1 by both TBBPA and TCBPA	[10]
				0/3.1/6.3/13/25/50/100 µM TBBPA/TCBPA +10 nM T3	With T3, inhibition of transcriptional activities by TBBPA and TCBPA/	
Mouse	Cerebellum (C17.α cells)	Luciferase reporter gene assay	24 h	0/10^−9^–10^−5^ M TBBPA ±0.1/1/10 nM T3	Antagonistic effect at least in part independent from T3 concentration.	[64]
Mouse	Oligodendrocyte precursors cells	Stimulation/inhibition	48 h	0/10^−5^ M BPA ±100 nM T3	No variation in TRα levels; TRβ1 levels significantly decreased compared to controls.	[67]
Rat	Adrenal medulla pheochromocytoma (PC12 cells)	Luciferase reporter gene assay	16 h	0/10/20/40/60/100 µM TBBPA ±1/100 nM T3	Agonistic activity in the absence of T3. With 1 nM T3, antagonistic activity counteracted by large excess of T3.	[65]
Rat	Thyroid pituitary tumor (GH3 cells)	Luciferase reporter gene assay	24 h	0–500 µM BPA/0–100 µM TBBPA/TCBPA ±0.25 nM T3	With T3, slight induction of luciferase activity at doses up to 1 µM. Without T3, no effect from TBBPA and TCBPA.	[76]
				0.25 nM T3 ±0/0.5/1/5/10/15 µM AM	AM induced T3-mediated response up to 1 µM.	
Rat	Thyroid pituitary tumor (GH3 cells)	Luciferase reporter gene assay	24 h	0/0.1/1/5/10/50 µM BPA/BPS/BPF ±1 nM T3	Agonistic activity of all chemicals on TH signaling in the absence of T3 and of BPA and BPF in the presence of T3.	[80]
African green monkey (*Cerchopitecus aethiops*)	Kidney (CV-1 cells)	Luciferase reporter gene assay	24 h	10^−6^–10^−4^ M TBBPA/TCBPA/BPA +10 nM T3	With T3, antagonistic activity in cells transfected with TRβ1. In the absence of T3, no effects of the three chemicals.	[73]
African green monkey (*Cerchopitecus aethiops*)	Kidney (CV-1 cells)	Luciferase reporter gene assay	n.d.	10^−9^–10^−7^ M BPA ±0.1 nM T3	With T3, suppression of TR-mediated transcription also in the presence of SRC1. No effects of BPA on transcription in the absence of T3.	[77]
		Mammalian two-hybrid assay	n.d.	10^−8^ M BPA ±0.1 nM T3	No effects of BPA on T3-mediated binding of SRC1 to TRβ1.	
				10^−9^–10^−7^ M BPA ±0.1 nM T3/±10 nM T4	Transcription activated by increasing concentrations of BPA in the presence of both T3/T4 and NCor/SMRT1.	
				10^−8^ M BPA ±0.1 nM T3/±10 nM T4	Overexpression of either β-integrin or c-Src reduced recruitment of N-CoR or SMRT to TR-β1 stimulated by BPA in the presence of T3/T4.	
Human	Hepatoblastoma (HpeG2 cells)	Luciferase reporter gene assay Mammalian two-hybrid assay	24 h	10^−9^/10^−7^/10^−5^ M BPA +10 nM T3	With T3, dose-dependent inhibition of transcription mediated by native TRα1 and TRβ1.	[2]
			n.d.	10^−9^/10^−7^/10^−5^ M BPA ±1/3/6 nM T3	Enhancement of interaction of TRs with N-CoR in a dose-dependent manner.	
Human	Hepatocarcinoma (HepG2 cells)	Luciferase reporter gene assay	24 h	10^−11^–10^−5^ M TBBPA ±1 nM T3	Activation of expression in the absence of T3 and antagonistic effect with T3 at the same dose (10^−4^M).	[72]
		qRT-PCR	24 h	10^−5^ M TBBPA ±1 nM T3	Antagonistic effect on T3-induced *DIO1* expression.	
Human	Embryonic kidney (HEK293 cells)	Luciferase reporter gene assay	24 h	0, 10^−9^–10^−5^ M TBBPA ±0.1/1/10 nM T3	Antagonistic effect stronger at lower T3 concentration.	[64]
***Cell Proliferation***						
Rat	Thyroid pituitary tumor (GH3 cells)	WST-1 cell proliferation assay	48/96 h	10^−9^–10^−6^ M BPA/BPAF/BPAP/BPB /BPC/BPF/BPM/BPP/BPS/BPZ ±6.4*10^−10^ M T3-EC_50_	All BPA analogues alone stimulated cell proliferation at the highest concentration. BPAF had the strongest effect. With T3, inhibition of cell proliferation at 48 h; agonistic effects at 96 h by high doses of BPA, BPAF, BPF, BPS, BPZ.	[85]
				10^−9^–10^−6^ M BPA/BPAF ±10^−12^ M E_2_/±6.4*10^−10^ M T3-EC_50_	Additive like-effects of co-treatment with BPA analogues and E_2_. With T3, inhibition of analogues alone and enhancement of cell proliferation by co-treatment with E_2_.	
Rat	Thyroid pituitary tumor (GH3 cells)	WST-1 cell proliferation assay	7 days	10^−8^–10^−4^ M TTBPA/TCBPA ±0.1/1 nM T3	Stimulation of cell growth and, with T3, no inhibition of induction of GH3 cell growth.	[71]
		GH-production assay	48 h	10^−8^–10^−4^ M TTBPA/TCBPA	Stimulation of GH release from cells.	
Rat	Thyroid pituitary tumor (GH3 cells)	GH production assay	48 h	10^−8^–10^−5^ M BPA/TTBPA/TCBPA	Stimulation of GH release from cells by only TTBPA and TCBPA. With T3, no inhibition of cell growth.	[83]
Rat	Thyroid pituitary tumor (GH3 cells)	T-screen assay	n.d.	0/0.1/1/5/10/50 µM BPA/BPS/BPF ±1 nM T3 ±2 µM AM	For all chemicals, induction of cell proliferation only with T3, and inhibition of growth in the absence of T3. Agonistic actions of BPs were antagonized by AM.	[80]
Rat	Thyroid pituitary tumor (GH3 cells)	T-screen assay	6 days	10^−8^–10^−5^ M BPA/BPA-DM/TBBPA ±0.5 nM T3	Stimulation of growth by all tested chemicals. In presence of T3, potentiating effect on T3-induced growth.	[81]
				0.5/1 nM T3; 5*10^−7^–5*10^−6^ BPA/BPA-DM; 1*10^−5^/2.5*10^−5^ M TBBPA ±1 nM ICI	Suppression of induced cell proliferation by the antiestrogen ICI. None of the compounds able to counteract the inhibitory effects of ICI.	
Rat	Thyroid pituitary tumor (GH3 cells)	T-screen assay	96 h	10^−7^–10^−5^ M BPA/TBBPA/TCBPA ±0.25 nM T3	No effects on growth for all compounds in the absence of T3. With T3, BPA stimulated growth with maximum potentiation at 10^−6^M, then cytotoxicity.	[82]
Rat	Thyroid pituitary tumor (GH3 cells)	T-screen assay	96 h	10^−12^–10^−6^ M TBBPA ±0.25 nM T3	With T3, potentiation of T3-mediated cell growth. In the absence of T3, no effects on cell proliferation.	[84]
Human	PTC (BHP10-3 cells)	Cell Counting Kit-8	24/48/72 h	10^−8^–10^−3^ M BPA 10^−9^–10^−4^ M E_2_	Similar proliferative effects of BPA and E_2_ with non monotonic dose-response curve and progressive effects over time.	[86]
***Cytotoxicity***						
Zebrafish (*Danio rerio*)	Hepatocyte (ZFL cells)	Fluorescence (AlamarBlue^TM^ assay)	24/96 h	0/10–1000 µM BPA	24 h LC50 of BPA: 367.1 µM 96 h LC50 of BPA: 357.6 µM	[75]
				0/3.16/10 µM TBBPA	24 h LC50 of TBBPA: 4 µM 96 h LC50 of TBBPA: 4.2 µM	
Amphibian (*Xenopus laevis*)	XL58-TRE-Luc cells	Cell Count Reagent SF kit	48 h	0/0.5/1/2/4/8/16 µM BPA/TBBPA	Cell viability not compromised up to 4 µM for both chemicals.	[70]
Chinese hamster	Ovary (CHO-K1 cells)	EGFP fluorescence	24 h	0/3.1/6.3/13/25/50/100 µM TBBPA/TCBPA +10 nM T3	Cytotoxicity at the highest concentrations tested for all chemicals.	[10]
African green monkey (*Cerchopitecus aethiops*	Kidney (CV−1 cells)	MTT assay	24 h	1/10/20/50/100 µM TBBPA/TCBPA/BPA ±10 nM T3	Cytotoxicity of all chemicals at the highest concentration tested in the absence and in the presence of T3.	[73]
Rat	Adrenal medulla pheochromocytoma (PC12 cells)	MTT assay	16 h	100 µM TBBPA/TCBPA	No significant effects on cell viability.	[65]
Rat	Thyroid pituitary tumor (GH3 cells)	Fluorescence (AlamarBlue^TM^) assay	4 h	0–500 µM BPA/0–100 µM TBBPA/TCBPA ±0.25 nM T3	At doses >10 µM TBBPA and TCBPA or 100 µM BPA visible cytotoxicity.	[76]
Rat	Immortalized thyroid follicular cells (FRTL-5 cells)	MTT-assay	24/72 h	10^−9^–10^−4^ M BPA	No effects on cell survival at any dose at either 1 day or 3 days of treatment.	[79]
Human	Thyroid anaplastic carcinoma (Cal-62 cells)	MTT assay (on each cell type)	24 h	0/25/50/75/100/150/200 µM BPA/TBBPA	TBBPA EC_50_ Cal 62 cells: 200 µM A549 cells: 168 µM NRK cells: 52 µM No toxic effects of BPA in the tested concentration range.	[87]
Rat	Kidney epithelial (NRK cells)					
Human	Epithelial alveolar type II-like lung (A549 cells)	DNA synthesis by BrdU-assay (on each cell type)	24/48/72/ 96/120 h	0/25/50/75/100/150/200 µM TBBPA ±10 µM U0126	NRK cells the most sensitive to TBBPA (decreased growth at >10 µM). In Cal-62 cells, significant inhibition of growth after 24 exposure to TBBPA >100 µM or 10 µM U0126.	
***Competitive Binding With Thyroid Hormone Binding Proteins***						
Amphibian (*Xenopus* *laevis)*	XL58-TRE-Luc cells	TTR and TR competitive binding assay	1–1.5 h	10^−10^–10^−5^ M BPA/TBBPA +0.1 nM ^125^I-T3	Inhibition of T3 binding to TTR (TBBPA IC_50_ = 3.7+0.29 nM; BPA IC_50_ = 1670+30 nM) and, with weaker affinity, to TR.	[70]
Chinese hamster	Ovary (CHO-K1 cells)	Competitive binding assay	40 min.	10^−7^–10^−4^ M BPA/TTBPA/TCBPA +0.1 nM ^125^I-T3	Inhibition of binding to TR (IC_50_TBBPA = 3.5 µM; IC_50_TCBPA = 9.5 µM).	[10]
Rat	Thyroid pituitary (MtT/E-2 cells)	Competitive binding assay	40 min.	10^−7^–10^−4^ M TTBPA/TCBPA; 10^−5^–10^−4^ M BPA +0.1 nM ^125^I-T3	Inhibition of T3 binding to TR by TBBPA and TCBPA. Little effect by BPA.	[71]
Rat	Thyroid pituitary tumor (GH3 cells)	T4-TTR competition binding assay	Overnight	1 nM–1 µM TBBPA + 55 nM (^125^ I-T4+T4)	Potent antagonism with T4 in TTR binding. IC_50_ = 0.031 µM.	[85]
Rat	Liver microsomes	T4-TTR competition binding assay	Overnight	1.95–500 nM TBBPA/TCBPA + 55 nM (^125^I-T4+T4)	For TBBPA, maximum displacement (96.5%) of T4 from TTR at 500 nM. IC_50_ = 7.7 ± 0.9 nM	[92]
***Perturbation of Thyroid Hormone Uptake***						
Dog	Kidney tubule (MDCK cells)	MCT8HTS assay	10 min.	2/4/8/18/32/62/125/250 µM BPA + 3.3 µM T3	Inhibition of T3 uptake mediated by MCT8.	[94]
Mouse	Primary astrocytes	Nonradioactive uptake assay	15 min.	10 µM BPA + 10 µM T3	Decrease in MCT8-mediated T3 uptake.	[98]
***Dysregulation of Gene Expression***						
Zebrafish (*Danio rerio*)	Hepatocyte (ZFL cells)	qRT-PCR	24 h	10/25/50% LC50 BPA/TBBPA	Inhibition of expression of *Dio1, Dio3, Trb, Sult1-st1, -st2, -st3, -st-5* and *Ugt2a1*, and induction of *Ugt1ab* genes by BPA. Inhibition of expression of *Dio3,* and up-regulation of *Ttr, Sult1-st2, sult1-st5,* and *Ugt2a1* by TBBPA.	[75]
Amphibian (*Xenopus laevis)*	Tadpole tail culture	Semi-quantitative RT-PCR	5 days	10^−7^–10^−5^ M BPA ±2.5*10^−8^/10^−7^/4*10^−7^ T3	Inhibition of *Trα* and *Trβ* mRNA expression. T3 counteracts the inhibitory effects of BPA on *Trα* and *Trβ* mRNA expression dose-dependently.	[66]
Rat	Liver and brown adipose tissue	Deiodinase 1 assay	1 h	0/0.005/0.05/0.5/5 mmol/L BPA	Inhibition of both hepatic DIO1 and brown adipose tissue DIO2 activities, with a greater effect on DIO1. IC_50_ (D1 activity) = 0.183 mmol/L IC_50_ (D2 activity) = 1.11 mmol	[102]
		Deiodinase 2 assay	3 h			
Rat	Thyroid pituitary tumor (GH3 cells)	qRT-PCR	48 h	10^−9^–10^−6^ M BPA/BPAF ±10^−12^ M E_2_	Inhibition of transcription of *Tshβ, Trα,* and *Trβ*, *Dio1* and *Dio2*. With E_2_, a more pronounced decrease of *Trα, Trβ* and *Dio2*, and increase of *Tshβ* transcription	[85]
Rat	Immortalized thyroid follicular cells (FRTL-5 cells)	qRT-PCR	24 h	0/1/10/100 mg/LBPA/BPB/BPF/BPS 0/0.1/1/10 mg/L BPAF/BPAP/BPC/BPM/BPP/BPZ	Stimulation of transcription of *Tsh-r, Pax8, Nkx2-1, S lc5a5, Tg, Tpo* by BPA, BPAF, BPAP, BPM, BPS.	[99]
	Thyroid pituitary Tumor (GH3 cells)		48 h	0/0.01/0.1/1/10 mg/L BPA/BPAF/BPAP/BPB/BPC/ BPF/BPM/BPP/BPS/BPZ	Inhibition of transcription of *Tshβ, Trα,* and *Trβ*, and *Dio2* by BPA, BPF, BPM, and BPZ. Some analogues but not BPA down-regulated *Dio1.*	
Rat	Immortalized thyroid follicular cells (FRTL-5 cells)	qRT-PCR	6/24/48 h	0/10/30/100 µM BPA	Up-regulation of *Tg, Pax-8, Foxe-1* and down-regulation of *Slc5a5, Tpo,* and *Nkx2-1* transcripts at the highest dose.	[100]
		Iodine uptake assay	1 h	10^−7^–10^−4^ M BPA +10 µM NaI	Concentration-dependent decrease of iodine uptake.	
			24/48 h	0/10/30/100 µM BPA	Significant decrease in iodine uptake at non-cytotoxic doses of BPA in the absence of NaI	
Rat	Immortalized thyroid follicular cells (FRTL-5 cells)	Microarray analysis/ qRT-PCR	1/3/7 days	10^−9^ M BPA	Deregulation of 372 and 1041 genes after 3 and 7 days, respectively. Most genes had a fold change >2 at both time points. Following exposure longer than 7 days, inhibition of genes involved in the DNA replication and repair network.	[101]
		Alkaline comet assay/ TUNEL assay	28 days + 5 days UV	10^−9^ M BPA	After irradiation at 48 and 96 h, higher content of DNA damage in the BPA-treated cells. Until 120 h post irradiation, higher apoptotic levels in the BPA-treated cells.	
Rat	Immortalized thyroid follicular cells (FRTL-5 cells)	Luciferase reporter gene assay	24 h	10^−15^–10^−4^ µM BPA ±1.78/3.44/6.87/13.7/27.5 ng/µl TSH	Enhancement of *Tg*-promoter activity also in the presence of the highest dose of TSH.	[89]
				10^−9^ M BPA ±1 µM ICI/TAM	No effect of the two antiestrogen on the activity of *Tg*-promoter induced by BPA.	
Human	Ovary cells (SVKO3 cells)	qRT-PCR	24 h/72 h	10^−9^–10^−4^ M BPA	Increase of transcription levels of *Slc5a5, Tpo, Tsh-r, Tg, Pax8, Nkx2-1, Foxe 1.* Increase of transcription of *PAX8* also in SVKO3 cells.	

**Table 3:** Summary of in vivo studies analyzing effects of exposure to bisphenols on thyroid function.

Species	Method	Window of Exposure	Doses Tested	Principal Results	Reference
***Rodents***					
Male and female CD-1 mice	ELISA	PND28-PND56	0/10/100 mg/L BPA in water	Significant reduction of FT4 but not FT3 levels at both doses. No effects of sex on FT3 and FT4 levels.	[113]
Pregnant female rats (Crj: CD (SD) IGS strain)	Chemiluminescence immunoassay	GD6-PND20	0/4/40 mg/kg BW BPA per day by diet	In male and female offspring no significant variations in T4 levels at 1, 3, 9 weeks of age.	[108]
	TSH stimulation assay	Offspring at 9 weeks of age; 1 day treatment	TSH 25 mIU/5µL/g BW BPA intraperitoneally + 125 mIU 5µL/g BW BPA intramuscularly	In response to exogenous TSH, elevation of T4 levels, but any significant difference between control and BPA groups of both sexes.	
Pregnant female Sprague Dawley rats	Radioimmunoassay	GD6-PND20	0/1/10/50 mg/kg BW BPA per day by diet	Increase of TT4 levels in both male and female pups only on PND15. No effects on serum TSH in male pups on PND15 among the different treated-groups.	[104]
Pregnant female Sprague Dawley rats	Electrochemiluminescence immunoassay	GD10-PND20	0/100/1000/10,000 ppm TBBPA by diet	In male pups on PND20, dose-unrelated, but statistically significant decrease of serum T3 levels whereas no significant variations of T4 and TSH. At PNW11, any changes of THs levels in any groups.	[107]
	Necropsy			No significant changes in thyroid weights of treated dams and offspring compared with controls but dose-unrelated increased thyroid weight in all treated groups of dams.	
	Histology			No significant increased incidence of diffuse thyroid follicular cell hypertrophy in dams at the highest dose.	
Pregnant female Sprague Dawley rats (strain code 23)	Radioimmunoassay	GD6-PND15	0/2.5/25/250/2500/25,000 μg/kg BW BPA per day by diet	No effects on T4 and TRH levels in male and females pups on PND15.	[109]
Pregnant female Sprague Dawley rats	ELISA	GD11-PND21	0/0.1/50 mg/L BPA per day in water	In dams, at 0.1 mg/L reduction of FT4 levels at delivery and on PND7. In male pups on PND7, increased FT4 levels at 0.1 mg/mL and decreased FT4 levels at 50 mg/L. In females no effects at each dose at all days.	[112]
Sprague Dawley rat dams	Radioimmunoassay	GD6-PND15 GD6-PND21 GD6-PND90	2.5–2700/100,000/300,000 μg/kg BW BPA per day by diet	No effects on THs levels, thyroid weight, or thyroid histology in the “low-dose” region. On PND15, elevation of T3 at both high doses. On PND90, increase of TSH in females at both high doses and of T4 in males at the highest dose.	[118]
Female Sprague Dawley rats	ELISA	GD11-PND21	0.1 mg/L BPA per day in water	In dams, after 10 days from exposure, significant decrease in TT4 and FT4 levels, but no variation of TT3 and FT3 levels. On PND21 but not on PND90, significant decrease of TT4, TT3, FT4, and FT3 levels in male pups.	[111]
Female Sprague Dawley rats	Radioimmunoassay/ELISA	PND1-PND10	0, 5 μg/50μL BPA (B5), 50 μg/50 μL BPA (B50), 500 μg/50 μL L BPA (B500) in castor oil	On PND13, no differences in TSH levels. In estrus, on PND90, increased TSH levels following exposure to B50, but not B5 or B500. In adult females, lower T4 levels at B5 and B500, but not B50. No significant differences of T3 among groups.	[117]
Neonatal males *R. norvegicus* (Wistar strain)	ELISA	PND15-PND30	0/20/40 mg/kg BW/BPA per day by diet	On PND30, significant elevation of TSH levels, accompanied by a notable reduction of T3, T4, and GH levels in a dose-dependent manner.	[116]
Male and female CD^®^ rats	Electrochemical luminescence immunoassay	13 consecutive weeks. 6-week recovery period of animals for both control and 1000 mg/kg/ day groups.	0/100/300/1000 mg/kg BW TBBPA per day by diet	No effects on TSH and T3 levels at any dose or time in both sexes. Decrease of T4 levels at all doses in both males and females. Following recovery period, T4 levels in males but not in females at 1000 mg/kg were comparable to controls.	[110]
	Necropsy/Histology			No effects of treatment on thyroid weight and histopathology	
Wistar rats dams	Radioimmunoassay	GD1-PND21	0/10 (BPA10)/50 μg/kg BW per day (BPA50) by diet	On PND21, no change in THs levels in dams and in female pups, whereas in the BPA10 group of male pups lower T3 levels without any variation in T4 levels. On PND180, decrease of T3 levels in females and T4 levels in males among the BPA10 group.	[115]
Adult female Wistar rats	Radioimmunoassay	15 days	40 mg/kg BW per day BPA by diet	Higher T4 levels in BPA-exposed animals, whereas no variations of T3 levels.	[106]
	Thyroid iodine uptake/TPO activity			Significant reduction of TPO and NIS activity.	
	ROS generation			In BPA-treated animals, significant generation of H_2_O_2_ generation in the thyroid.	
	qRT-PCR			Significant reduction of *Tshβ* mRNA levels in the pituitary of the treated rats.	
Adult male Wistar rats	RIA	15 days	0/40 mg/kg BW/BPA per day by diet	After 15 days of treatment, significant reduction of liver DIO1 activity, whereas no effects on brown adipose tissue DIO2 activity. Significant increase of TT4 levels but no variations of TT3 levels. Significant reduction of T3/T4 ratio.	[102]
Wistar rats (HsdCpb:WU) of both sexes	Radioimmunoassay	70 or 14 days before mating – after mating (males) or PND21 (females) 28 days	0/3/10/30/100/300/1000/3000 mg/kg BW TBBPA per day by diet (reproduction study) 0/30/100/300 mg/kg BW TBBPA per day by diet (subacute toxicity study)	In the reproduction study, decrease of T4 levels in pups of both sexes, and increase of T3 levels only in females. In the subacute toxicity study, significant decrease of T4 and increase of T3 levels in males, whereas in females parallel though not significant trends.	[114]
	Necropsy			Dose-dependently increase of pituitary weight in male pups in the reproduction study. No effects in the subacute toxicity study.	
	Histology			No changes observed in the histology of the pituitary gland both in the reproduction and in the subacute toxicity studies.	
Female F344 rats	Necropsy	64 weeks	250/1000 μg/kg BW per day BPA by diet ±2800 mg/kg sc. DHPN ±1000 μg/L KI in water	In the group exposed to DHPN, statistical significances among all groups, and the KI group had the heaviest thyroid weights. In the group not exposed, no significant differences were found among groups.	[105]
	Histology			In the group exposed to DHPN + KI + 1000BPA, all thyroids had a tumor or focal hyperplasia. Significant difference in the total number of hyperplasia lesions among all groups of animals exposed to DPNA.	
	Chemiluminescence immunoassay/ELISA			In the groups exposed to DHPN, TSH was significantly higher in the KI group than in the controls and the highest FT4 concentration was in the BPA1000 group. In the groups not exposed to DHPN, the highest concentration of TSH was in the controls whereas FT4 increased with increasing doses of BPA.	
	Western blotting detection			In the groups exposed to DPNA, increased protein levels of ERα in the BPA250 and BPA1000 groups compared to the control.	
***Sheep***					
Lacaune ewes	Radioimmunoassay	GD28-GD128	0/0.5/50/5000 μg/kg per day sc. BPA	In mothers, no effects on TT4 and TSH levels, but significant decrease of FT4 (at the lowest dose) and TT3 (at the lowest and middle doses) throughout pregnancy. No changes in TT4 and FT4 levels in fetal jugular blood on GD132-GD134.	[123]
Lacaune ewes	Radioimmunoassay	GD28–GD145	0/5 mg/kg BW per day sc BPA	In pregnant ewes, reduction with time of TT4 but not of FT4. Decrease of jugular blood TT4 and FT4 concentration within the 1^st^ hour of life and of TT4 concentration in cord blood. Slight decrease of TT3 in the 1^st^ hour of life. At 2 months of life no changes in THs levels.	[122]
***Zebrafish***					
GFP-positive transgenic zebrafish embryos	Fluorescence	24–48hpf/24–72 hpf	10^−7^/5×10^−7^/10^−6^/5*10^−6^/10^−5^ M BPA ±10^−8^ M T3	In the presence of T3, the two highest doses of BPA inhibited T3-induced transcriptional activity during 48 h exposure. No effects of BPA or T3 in the presence of T3 during 24h exposure.	[126]
Wild-type zebrafish (*Danio rerio*) embryos	qRT-PCR	72 h	0.01/0.1/1.0 μM BPA/BPS/TBBPA/TBBPS	At 0.1 μM, BPA and TBBPS up-regulated *Trβ* transcripts whereas BPS and TBBPS showed no significant effect. At 1.0 μM BPS and TBBPS up-regulated *Trβ* mRNA levels, while BPA had a down-regulating effect.	[68]
Wild-type zebrafish (*Danio rerio*) embryos	qRT-PCR	24–48 hpf	10^−8^–10^−6^ M BPA	Induction of *Tg* expression at 10^−8^ and 10^−6^ M. Up-regulation of *Pax8* transcripts at low and high doses and of *Pax2a* and *Tsh* transcripts only at high dose.	[89]
Wild type zebrafish (*Danio rerio*) embryos	ELISA qRT-PCR	2–168 hpf	0/5/50/500 μg/L BPAF	Significant decrease of TT3, FT4 and TT4 levels at 50 and 500 μg/L. Reduction of FT3 levels in all treated groups.	[13])
				Up-regulation of *Tshβ* at 50 μg/L and significant decrease of transcription at the highest dose. Up-regulation of *Tg* and *Dio2* at 50 μg/L, *Dio1* at 50 and 500 μg/L, and *Ttr* at all doses. Down-regulation of transcription of *Trβ* at 50 μg/L, *Slc5a5* and *Trα* at 50 and 500 μg/L.	
Wild type zebrafish (*Danio rerio*) embryos	ELISA	75 dpf	0/0.1/1/10 and 100 μg/L BPS	Lower levels of T3 and T4 in males exposed to 10 and 100 μg/L. Lower levels of T3 and T4 in females exposed to 100 μg/L.	[131]
Wild-type zebrafish (*Danio rerio*) embryos-larvae	ELISA	<4–120 hpf	0.08/0.4/2 mg/L BPA/BPF; 2/10/50 mg/L BPS; 0.04/0.18/0.68 mg/L BPZ	Significant increase of T3 levels at 0.4 mg/L BPA and at 50 mg/L BPS (with a dose-response trend) and of T4 at 2.0 mg/L BPF (with a dose-response trend).	[37]
	qRT-PCR		0.4<72 mg/L BPA or BPF; 10/50 mg/L BPS; 0.08/0.4 mg/L BPZ	Up-regulation of *Hhex, Tg, Ttr, Dio1, Ugt1ab* genes by BPA. Up-regulation of *Hhex, Ugt1ab* genes by BPF, of *Crh, Tshβ, Tsh-r, Hhex, Tpo, Ttr, Ugt1ab* by BPS, and of *Tshβ* by BPZ.	
Wild-type zebrafish (*Danio rerio*) embryos-larvae	ELISA	2–144 hpf	0.2/2/20/200 µg/L BPF	Dose-dependent decrease of TT4 levels in BPF treated groups. Significant elevation of TT3 at 200 µg/L and TSH at 20 and 200 µg/L BPF.	[38]
	qRT-PCR			Up-regulation of *Crh* and *Tg* at 2,20, 200 µg/L. Induction of transcription of *Slc5a5, Dio2, Ugt1ab* at 20 and 200 µg/L. Down-regulation of *Ttr* at 20 and 200 µg/L.	
Wild-type zebrafish (*Danio rerio*) embryos-larvae	qRT-PCR	0/1/5 dpf; exposure for 24/48h	10^−5^ M BPA ±10^−8^ M T3	Weak effects of BPA alone on gene expression compared with controls, except for the induction of TSH expression in the group 1day/48 h. With T3, reduction of *Trα, Trβ*, and *Tsh* expression in 1-day-old embryos at both 24 and 48 h, but up-regulation of *Trα* and *Trβ*, by 24-h exposure in 0-day-old embryos and of *Tsh* by 48-h exposure in 5-day-old larvae.	[126]
Wild-type zebrafish (*Danio rerio*) embryos-larvae	ELISA	2–144 hpf	50/100/200/400 μg/L TBBPA	Dose-dependent increase of T4 levels and dose-dependent decrease of T3 levels.	[128]
			200 μg/L TBBPA/20 μg/L T3/200 μg/L TBBPA+20 μg/L T3	After exposure to T3, significant decrease of T4 and significant increase of T3 contents. After co-treatment TBBPA and T3, decrease of T4 and increase of T3 contents, which were significantly different from the control group (only for T3 increase) and the TBBPA treated group.	
	qRT-PCR		50/100/200/400 μg/L TBBPA	Up-regulation of *Tsh* and *Tg* and down-regulation of *Ttr* and *Trβ* transcript levels.	
			200 μg/L TBBPA/20 μg/L T3/200 μg/L TBBPA+20 μg/L T3	After exposure to T3 and TBBPA + T3 significant up-regulation of *Tsh* and *Tg* whereas no changes in *Ttr* and *Trβ* mRNA levels.	
Wild-type zebrafish (*Danio rerio*) embryos-larvae	ELISA	2–168 hpf	1/3/10/30 μg/L BPS	Significant decrease of TT4 levels at 10 and 30 μg/L and TT3 levels at the highest dose. Significant increase of TSH in the 10-, and 30 μg/L exposure groups.	[129]
	qRT-PCR			Up-regulation of *Crh, Tg, Dio1,* and *Ugt1ab* at 10 and 30 μg/L. Induction of transcription of *Pax8, Slc5a5, Tg,* and *Dio2* at 30 μg/L. Down-regulation of *Ttr* mRNA at 3, 10, and 30 μg/L. No effects on transcription of *Trα, Trβ,* and *Dio3.*	
Wild-type zebrafish (*Danio rerio*) embryos-larvae	qRT-PCR	96–192 hpf	0%/10%/50%/75% of the 96 h -LC50 BPA/TBBPA	Induction of *Trα, Tshβ,* and *Ttr* in larvae and of *Trα* and *Ttr* transcripts in embryos by TBBPA. Down-regulation of *Trβ* and *Tshβ* mRNA in embryos by TBBPA. Up-regulation of *Tshβ* mRNA in larvae by BPA. No effects on *Slc5a5* and *Tg* expression in larvae neither on *Tpo* in embryos.	[133]
Wild type zebrafish (*Danio rerio*) larvae	qRT-PCR	2–120 hpf	0/100/200/300/400 μg/L TBBPA	Slight up-regulation of *Dio1, Trβ,* and *Tsh,* and significant up-regulation of *Trα* at low doses. Significant down-regulation of *Tpo*. No significant effects on expression of *Dio2* and *Dio3* transcripts.	[132]
Wild-type adult zebrafish (*Danio rerio*)	qRT-PCR	72 h	50 μg/L/100 μg/L BPA or BPS	After BPA exposure, increase of *Slc5a5* mRNA levels at both doses and *Tpo* transcript levels at the lowest dose. No effects on *Tg* expression. After BPS exposure, increased expression of *Slc5a5* and *Tg* transcripts at the highest dose and *Tpo* transcripts at the lowest dose.	[135]
Adult male zebrafish (*Danio rerio*)	ELISA	21 days	0/24.7 µg/L BPAF/5.6 µg/L SMX/24.7 µg/L BPAF + 5.6 µg/L SMX	Slight decrease of T4 levels in fish exposed to BPAF. Significant increase of T4 in fish exposed to the mixture BPAF + SMX.	[134]
	qRT-PCR			Up-regulation of *Trh, Trhr, Tshβ, Dio2* and down-regulation of *Tpo, Slc5a5, Tg* mRNA levels in fish exposed to BPAF. Up-regulation of *Trα, Trβ, Dio1, Dio2, Tpo, Tg, Slc5a5* and down-regulation of *Trh, Trhr, Tshβ* transcript in fish exposure to mixture.	

**Table 4:** Summary of human studies on the association between bisphenols exposure and thyroid parameters.

Study Design	Country	Study Sample	Sample Size (N)	Age	Principal Results	BPA Concentration	Confounders	Reference
Prospective	Japan	Women with a history of three or more (3–11) first-trimester miscarriages. Blood samples collected 5–9 days after ovulation in at least two cycles.	45 patients	27–36	No difference in serum BPA levels between patients with and without hypothyroidism. Serum TSH levels not estimated.	Patients with hypothyroidism: Mean (SD): 2.99 ± 3.04 µg/L Patients without hypothyroidism: Mean (SD): 2.50 ± 5.70 µg/L	-	[151]
Prospective	Belgium	Overweight and obese individuals. Lean individuals. For obese individuals, urine samples collected at baseline, before starting a weight loss program (N = 151) and after 3 (N = 95), 6 (N = 53), 12 months (N = 39).	151 obese individuals 43 lean individuals	≥18	The obese group had higher urinary levels of BPA. Positive relationship of urinary BPA with serum TSH in lean subjects.	*Obese individuals* 0—median: 1.7 µg/L 3 months—median: 2.0 µg/L 6 months—median: 1.7 µg/L 12 months—median: 1.5 µg/L *Lean individuals* Median: 1.2 µg/L	[1,7,34]	[152]
Prospective birth cohort	USA	Pregnant women from the CHAMACOS study. Urine samples collected at 12 and 26 weeks of gestation.	476	≥18	In mothers, no association of urinary BPA (average) with FT4 and TSH levels, and negative association (BPA 26 week) with serum TT4. Inverse association between maternal BPA (average, 26 week) and TSH in male newborns but not in females.	LOD: 0.4 μg/L Mothers: GM: 1.3 µg/g cr Median: 1.2 µg/g cr	[1,2,4,8,10,12,13,14,15,16,17,18,19,20,21]	[143]
Prospective birth cohort	USA	Pregnant women from the HOME study. Urine samples collected at both 16 and 26 weeks of gestation (N = 237).	249	≥18	Neither association of maternal BPA (16 week) with maternal THs or TSH levels nor of maternal BPA (average, 16 week, or 26 week) with THs or TSH levels in newborns. Significant inverse association of maternal BPA with TSH levels (average and 26 week BPA) and slight positive association with TT3 levels (26 week BPA) in females. Stronger relationship of BPA–TSH among girls born from iodine-deficient mothers.	LOD: 0.4 μg/L 16 weeks GM: 2.0 (95%CI 1.8–2.2) µg/g cr 26 weeks GM: 2.3 (95%CI 2.1–2.5) µg/g cr	[1,2,8,9,10,15,16,17,19,20,22,23,24,25]	[148]
Prospective birth cohort	Japan	Pregnant women at 23–35 weeks of gestation (singleton babies). Cord blood obtained at delivery.	283	≥18	No association between BPA concentration in cord blood and TSH or FT4 levels in newborns of both sexes.	LOQ: 0.04 µg/L GM: 0.051 µg/L IQR: <LOQ–0.076 µg/L	[19,38]	[146]
Nested case-control	USA	Women who delivered preterm (< 37 weeks of gestation) and controls of women who delivered a singleton infant after 37 weeks of gestation. Urine samples collected at up to 4 visits (median for each visit: 9.64, 17.9, 26.0, and 35.1 weeks of gestation).	116 cases 323 controls	≥18	IQR increase in BPA concentrations across study visits was significantly associated with lower TSH and higher FT4 levels. No effect on FT3, and inverse but not significant association of BPA with TT4 levels. No association of BPA with serum FT4 at visit 3. Significant inverse association of BPA with TSH levels at visits 3 and 4 and a slight increase of serum TT3 at visit 4.	Total: GM ± SD: 1.18 ± 2.82 µg/L 1 visit: GM ± SD: 1.33 ± 2.84 µg/L 2 visit: GM ± SD: 1.04 ± 2.85 µg/L 3 visit: GM ± SD: 1.22 ± 2.84 µg/L 4 visit: GM ± SD: 1.12 ± 2.70 µg/L	[2,3,4,8,17,19,25,35,36,37],	[139]
Case-control	Korea	Infants with congenital hypothyroidism and their mothers; healthy infants with their mothers. Blood samples collected.	26 congenital hypothyroidism mother–infant pairs 12 normal mother–infant pairs	<24 months	TBBPA levels not significantly different in the two infant groups. No significant correlation between TBBPA and any of the THs levels in infants with congenital hypothyroidism. In their mothers, positive weak correlation of TBBPA with serum FT4 and thyroid stimulating immunoglobulin.	*Normal group and their mothers* TBBPA mothers: 10.93 ng/g lipid TBBPA infants: 77.65 ng/g lipid *Congenital hypothyroidism group and their mothers* TBBPA mothers: 8.89 ng/g lipid TBBPA infants: 83.4 ng/g lipid	[1,47,48,49]	[140]
Case-control	Cyprus (n = 122) Romania (n = 90)	Females with thyroid nodules (diameter >3mm). Females without nodules. Two spot urine samples collected in Cyprus (7 day apart from each other). In Romania one spot sample collected.	212: 106 cases 106 controls	≥18	In the whole study population, median TSH and BPA levels were significantly lower in the cases. Significant positive association of BPA with TSH levels and TNs. Neither association of BPA, BPF, or ClxBPA with FT4 levels nor of BPF and ClxBPA with serum TSH. No association of BPF and ClxBPA with TNs.	LOD (BPA): 10 μg/L LOD (BPF): 13 μg/L LOD (ClxBPA): 10–17 μg/L Median: BPA: 2.25 (1.10–4.61) μg/L BPF: 0.46 (0.32–0.72) μg/L ClxBPA: 0.16 (0.15–0.19) μg/L BPA Cases: 1.75 (1.11–3.56) μg/L BPA Controls: 2.71 (1.08–5.91) μg/L	[1,3,10,11,39,40,41]	[147]
Case-control	Turkey	Children with HT. Children without HT. Spot urine samples collected.	29 cases (25 females and 4 males) 29 controls	8–16	No significant difference in urinary BPA levels between the two groups. Significant negative correlation between BPA and FT4 levels in HT group. No correlation between urinary BPA concentration and TPOAb levels.	LOD: 0.5 ng/mL Mean (± standard error of the mean) *Cases*: 7.31 ± 1.46 µg/g cr *Controls*: 7.72 ± 1.74 µg/g cr	-	[155]
Case-control	China	Women with TNs. Women without nodules. First morning urine collected.	1416: 705 cases 711 controls	≥18	Urinary BPA was significantly higher in cases than in controls. Increased prevalence of TNs with increasing urinary BPA quartiles. Significant association between urinary BPA and TNs in all age groups and in the thyroid autoantibody positive group.	LOD: 0.1 µg/L Median: *Total*: 3.08 (1.60–5.81) µg/g cr *Cases*: 3.27 (1.77–6.53) µg/g cr *Controls*: 2.92 (1.44–5.27) µg/g cr	[1,3,8,10,11,29,30,31,44,45,46]	[159]
Multicentre, cross-sectional	Italy	Patients with TNs. Patients with DTC. Blood collected from each patient.	27 with TNs 28 with DTC	≥18	Significant correlation between urinary BPAF concentration and risk of DTC in patients with TNs. BPS and BPB concentrations higher in patients with TNs, as compared with DTCs. TSH levels higher in patients with DTCs and in subjects exposed to BPE and BPA.	*Subjects with TNs* (median ± SD) BPAF: 12.47 ± 0 ng BPS: 59.29 ± 50.29 ng BPB: 29.92 ± 35.60 ng *Subjects with DTC* (median ± SD) BPAF: 9.44 ± 5.41 ng BPS: 34.79 ± 41.86 ng BPB: 14.36 ± 15.31 ng	[50]	[161]
Cross-sectional	USA	Men who are partners of subfertile couples. Single spot urine (N = 167), second (N = 75), and third (N = 4) urine samples collected.	167	18–55	Inverse relationship of BPA with TSH levels. No effects on T3 and T4 levels.	LOD: 0.4 µg/L GM and median: 1.3 µg/L	[1,2,3,4,5,6]	[150]
Cross-sectional	USA	Adult and adolescents from the NHANES 2007–2008. Single spot urine collected.	1346 adults 329 adolescents	≥20 12–19	In adults, inverse relationship of BPA with TT4 levels. Inverse trends between BPA quintiles and both TT4 and TSH.	GM: 2.03 µg/g cr GM: 1.88 µg/g cr	[1,2,3,7,8,9,10]	[145]
Cross-sectional	USA	Adults and adolescents from the NHANES 2007–2008. Urine sample collected.	710 females; 850 males	12–85	Negative association between urinary multiple EDCs including BPA and TT4 levels in males but not in females. Positive but not statically significant association of EDCs with T3 levels in females.	LOD (BPA): 0.13 µg/L Males: GM: 2.19 (95% CI 1.94–2.47) µg/L Females: GM: 2.00 (95% CI 1.80–2.23) µg/L	[1,2,4,9,10,11,26]	[142]
Cross-sectional	China	Workers in two semiautomatic epoxy resin factories. Spot urine collected at the end of the shift on Friday.	28 (21 males and 7 females)	22–62	The workers with the highest BPA concentrations (feeding position) had higher FT3, TT3, TT4 levels, and lower TSH levels. Urinary BPA significantly associated with higher FT3 levels (when office workers were excluded). Weak positive association between BPA and serum FT4.	*Total workers:* GM ± SD: 31.96 ± 4.42 μg/g cr *Feeding operators*: GM ± SD: 192.45 ± 2.78 μg/g cr *Crushing and packing:* GM ± SD: 17.08 ± 2.33 μg/g cr *Office workers:* GM ± SD: 11.60 ± 1.70 μg/g cr	[11]	[141]
Cross-sectional	China	Population-based study. Spot morning urine collected.	3394: 1354 males 2040 females	≥40	Significant positive association of urinary BPA with serum FT3 and inverse association with TSH levels both in men and in women. Positive association between high thyroid function and high urinary BPA levels.	LOD: 0.3 μg/L Median: 0.81 µg/L IQR: 0.47–1.43 µg/L *Overt and subclinical* *hypothyroidism subjects*: GM: 0.63 (95% CI 0.52–0.77) µg/L *Euthyroid subjects*: GM: 0.81 (95% CI 0.78–0.83) µg/L *Overt and subclinical hypothyroidism subjects*: GM: 1.05 (95% CI 0.84–1.32) µg/L	[1,3,4,8,11,17,27]	[149]
Cross-sectional	China	Students of primary schools. First morning urine samples collected.	718	9–11	BPA levels similar among boys and girls but increased with age. Significant inverse association between urinary BPA and thyroid volume. Risk of TNs increased with age without any association with sex or urinary iodine level. BPA inversely associated with risk of multiple TNs.	Urinary iodine: Median: 159 μg/L Urinary BPA: Median (IQR): 2.45 (1.09–5.97) µg/g cr	[1,3,7,11,42,43]	[158]
Cross-sectional	China	Patients with NG. Patients with PTC. Healthy controls. Spot blood and urine samples collected in the morning.	53 60 65	≥18	Urinary BPA and urinary iodine levels in NG and PTC groups were significantly higher than those in controls. Urinary BPA levels in the females of NG group and both the male and female PTC groups were higher than those in the control group of the same sex. Urinary BPA concentration was significantly lower in the female PTC group than in the female NG group. Urinary iodine in the PTC or NG groups was higher than that of controls of the same sex. Significant correlation between urinary BPA and iodine concentrations in all groups.	LOQ for BPA in urine: 0.1 µg/L LOQ for BPA in serum: 0.2 µg/L LOD for iodine in urine: 3 µg/L Serum BPA GM: 7.42 (4.03–13.82) µg/L Urinary BPA: GM: 2.82 (0.016–59.78) µg/g cr Urinary iodine: GM: 335.05 (71.25–2995.74) µg/g cr *BPA—Control group* GM: 1.06 (0.015–27.88) µg/g cr *BPA—NG group* GM: 5.22 (0.022–50.78) µg/g cr *BPA—PTC group* GM: 4.68 (0.032–29.30) µg/g cr	[11]	[160]
Cross-sectional	Thailand	Subjects from the National survey NHES 2009. Serum sample collected.	2340	≥15	Significantly inverse association of serum BPA with FT4 levels in males but not in females after exclusion subjects of thyroid autoantibodies. No association with serum TSH in both sexes.	LOD: 0.3 μg/L Median: 0.33 (0–66.91) µg/L	[1,3,7]	[144]
Cross-sectional	Thailand	Subjects from the National survey NHES 2009. Serum sample collected.	2361	≥15	Significant association of increasing BPA quartiles with positivity for TgAb and TPOAb both in men and women but not for TRab. Age, sex, and BMI were independent predictors of TgAb and TPOAb positivity.	LOD: 0.3 μg/L Median: 0.32 (0–66.9) µg/L	-	[156]

[1]: age; [2]: race and ethnicity; [3]: BMI; [4]: smoking; [5]: timing of collection of blood/urine samples by season; [6]: timing of collection of blood/urine samples by time of day; [7]: sex; [8]: education level; [9]: serum cotinine; [10]: urinary iodine; [11]: urinary creatinine; [12]: family income; [13]: country of birth; [14]: number of years spent in the United States; [15]: parity; [16]: gestational age at the time of blood collection; [17]: alcohol consumption; [18]: illegal drug use during pregnancy; [19]: newborn sex; [20]: delivery mode; [21]: age at the time of heel stick; [22]: prenatal vitamin use; [23]: Log_10_-PCB 153; [24]: delivery by Cesarean section; [25]: gestational week at delivery; [26]: menopausal status; [27]: occupation; [28]: total cholesterol; [29]: triglycerides; [30]: HDL-cholesterol; [31]: LDL-cholesterol; [32]: thyroglobulin antibody; [33]: thyroid peroxidase antibodies; [34]: weight loss; [35]: maternal age; [36]: health insurance provider, [37]: urinary specific gravity; [38]: days of mass screening test; [39]; thyroid hormones; [40]: study site; [41]: disease status; [42]: BSA; [43]: iodized salt consumption; [44]: total cholesterol; [45]: TgAb; [46]: TPOAb; [47]: obesity and other related diseases; [48]: medication; [49]: medical history; [50]: other EDCs.

## Abbreviations

3,3′-Cl2BPA	3,3′-dichlorobisphenol A
3,5-Cl2BPA	3,5-dichlorobisphenol A
95% CI	95% confidence interval
96 h-LC50	96 h median lethal concentration
AM	Amiodarone
BMI	Body mass index
BPA	Bisphenol A; 2,2-bis(4-hydroxyphenyl)propane)
BPAF	Bisphenol AF; 1,1,1,3,3,3-hexafluoro-2,2-bis(4-hydroxyphenyl)propane
BPAP	Bisphenol AP (4,4′-(1-phenylethylidene)bisphenol
BPB	Bisphenol B (2,2-bis(4-hydroxyphenyl)butane)
BPF	Bisphenol F; 4,4′-dihydroxydiphenylmethane
BPM	Bisphenol M; 4,4′-(1,3-phenylenediisopropylidene)bisphenol)
BPP	Bisphenol P (2,2-bis(4-hydroxyphenyl)butane)
BPS	Bisphenol S; 4,4′-sulfonyldiphenol
BPZ	Bisphenol Z; 1,1-bis(4-hydroxyphenyl)-cyclohexane
BPs	Bisphenols
BrdU	5′-bromo-2′-deoxyuridine
BSA	Body surface area
BW	Body weight
CHAMACOS	Center for the Health Assessment of Mothers and Children of Salinas
ClBPA	3-chlorobisphenol A
ClxBPA	Sum of ClBPA, 3,5-Cl2BPA, and 3,3′-Cl2BPA
cr	Creatinine
Crh	Corticotrophin-releasing hormone
c-Src/PI3K	Proto-oncogene tyrosine-protein/phosphatidylinositol 3-kinase
D1	Type 1 iodothyronine deiodinase
D2	Type 2 iodothyronine deiodinase
DHPN	N-bis (2-hydroxypropyl) nitrosamine
Dio1	Deiodinase 1
Dio2	Deiodinase 2
Dio3	Deiodinase 3
dpf	Days post-fertilization
DTC	Differentiated thyroid cancer
EC_50_	Median effective concentration
EDC(s)	Endocrine disrupting chemical(s)
EGFP	Excitation and emission of green fluorescent proteins
ELISA	Enzyme linked immunosorbent assay
ERs	Estrogen receptors
ERα	Estrogen receptor α
E_2_	17-β estradiol
ERK	Extracellular-signal-regulated kinase
Foxe1	Forkhead box E1
FT3	Free triidothyronine
FT4	Free thyroxine
GD	Gestation day
GFP	Green fluorescent protein
GH	Growth hormone
GM	Geometric mean
GPR30	G protein-coupled receptor 30
Hhex	Hematopoietically expressed homeobox
HOME	Health Outcomes and Measures of the Environment
hpf	hours post fertilization
HPT	Hypothalamic–pituitary–thyroid
HT	Hashimoto’s thyroiditis
IC_10_	Concentration at which 10% of the inhibition was observed
IC_50_	Concentration at which 50% of the inhibition was observed
ICI	Fulvestrant
IQR	Interquartile range
JNK	c-Jun NH2-terminal kinase
KI	Potassium iodine
LC_50_	Median lethal concentration
LOAEL	Low observed adverse effect level
LOD	Limit of detection
LOEC	Lowest observed effect concentration
LOW	Limit of quantification
MAPK	Mitogen-activated protein kinase
MCT	Monocarboxylate transporter
MEK	Mitogen-activated protein kinase/ERK kinase
MTT	3-(4,5-dimethylthiazol-2-yl)-2,5-diphenyltetrazolium bromide
NaI	Sodium iodide
N-CoR	Nuclear receptor co-repressor
n.d.	Not detected
NG	Nodular goiter
NHANES	National Health and Nutrition Examination Survey
NHES IV	Thai National Health Examination Survey
Nkx2-1	NK2 homeobox 1
OATP	Organic anion transport protein
Pax8	Paired box 8
PND	Postnatal day
PTC	Papillary thyroid cancer
qRT-PCR	Quantitative real time polymerase chain reaction
ROS	Reactive oxygen species
SAPK	Stress-activated proteins kinases
Slc5a5	Gene encoded for sodium iodide symporter
SMRT	Silencing mediator for retinoid and thyroid
SRC	Steroid receptor coactivator
Sult-st1/2/3/5	Sulfotransferase 1/2/3/5
T3	Triiodothyronine
T4	Thyroxine
TAM	Tamoxifen
TBBPA	Tetrabromobisphenol A
TBBPS	Tetrabromobisphenol S
TBG	Thyroxine binding globulin
TC	Thyroid cancer
TCBPA	Tetrachlorobisphenol A
Tg	Thyroglobulin
TgAb	Thyroglobulin antibody
TH(s)	Thyroid hormone(s)
TN(s)	Thyroid nodule(s)
Tpo	Thyroid peroxidase
TPOAb	Thyroid peroxidase antibody
TR(s)	Thyroid receptor(s)
TRAb	Thyroid receptor antibody
Trα	Thyroid receptor α
Trβ	Thyroid receptor β
TRH	Thyrotropin-releasing hormone
Trh-R	Thyrotropin-releasing hormone receptor
TSH	Thyroid stimulating hormone
Tshβ	TSH-specific β subunit
Tsh-r	Thyroid stimulating hormone-receptor
TTR	Transthyretin
TT3	Total triiodothyronine
TT4	Total thyroxine
Ugt	Uridine diphosphate -glucuronosyltransferase
